# Chemogenomic model identifies synergistic drug combinations robust to the pathogen microenvironment

**DOI:** 10.1371/journal.pcbi.1006677

**Published:** 2018-12-31

**Authors:** Murat Cokol, Chen Li, Sriram Chandrasekaran

**Affiliations:** 1 Axcella Health, Cambridge, Massachusetts, United States of America; 2 Laboratory of Systems Pharmacology, Harvard Medical School, Boston, Massachusetts, United States of America; 3 Faculty of Engineering and Natural Sciences, Sabanci University, Istanbul, Turkey; 4 Department of Biomedical Engineering, University of Michigan, Ann Arbor, Michigan, United States of America; University of Virginia, UNITED STATES

## Abstract

Antibiotics need to be effective in diverse environments *in vivo*. However, the pathogen microenvironment can have a significant impact on antibiotic potency. Further, antibiotics are increasingly used in combinations to combat resistance, yet, the effect of microenvironments on drug-combination efficacy is unknown. To exhaustively explore the impact of diverse microenvironments on drug-combinations, here we develop a computational framework—Metabolism And GENomics-based Tailoring of Antibiotic regimens (MAGENTA). MAGENTA uses chemogenomic profiles of individual drugs and metabolic perturbations to predict synergistic or antagonistic drug-interactions in different microenvironments. We uncovered antibiotic combinations with robust synergy across nine distinct environments against both *E*. *coli* and A. *baumannii* by searching through 2556 drug-combinations of 72 drugs. MAGENTA also accurately predicted the change in efficacy of bacteriostatic and bactericidal drug-combinations during growth in glycerol media, which we confirmed experimentally in both microbes. Our approach identified genes in glycolysis and glyoxylate pathway as top predictors of synergy and antagonism respectively. Our systems approach enables tailoring of antibiotic therapies based on the pathogen microenvironment.

## Author summary

The antibiotic resistance epidemic has created a pressing need to understand factors that influence antibiotic efficacy. An often-overlooked factor in the search for new treatments is the pathogen environment. Understanding the differences in pathogen sensitivity to antibiotics in lab conditions versus inside the host is necessary for translating new discoveries into the clinic. Hence, we experimentally measured the sensitivity of *E*. *coli* to drugs and drug combinations in different metabolic conditions. Our data revealed that the environment dramatically changes treatment potency. Each antibiotic class was affected uniquely by each metabolic condition. The large number of metabolic conditions inside the host greatly complicates the identification of effective therapies. To address this challenge, we present a computational approach called MAGENTA that accurately predicted efficacy of antibiotic regimens in different conditions, which we confirmed experimentally. Furthermore, we show that MAGENTA can be applied to other bacterial pathogens such as A. *baumannii* and *M*. *tuberculosis* without the need for generating expensive data in each organism. MAGENTA accurately predicted efficacy in the pathogen *A*. *baumannii* using data from *E*. *coli* by identifying genes that are common between the two bacteria. Our study revealed the significant yet predictable impact of environment on drug combination potency.

## Introduction

The threat of antibiotic resistance coupled with a diminishing pipeline of new drugs has created a pressing need to enhance efficacy of existing antibiotics [1]. Combinations of antibiotics are now being increasingly used to enhance the efficacy of treatment regimens and concurrently reduce resistance [2]. A key factor influencing the efficacy of antibiotic therapies is the environmental context [3,4]. Antibiotics need to act in diverse and complex metabolic environments *in vivo*, in contrast to well controlled lab conditions. Environmental factors such as the availability of oxygen and extracellular metabolites impact cell killing by antibiotics [5]. The strong impact of metabolic state on drug efficacy has been observed across diverse microbial pathogens [3,6,7].

In addition to individual drugs, pathogen metabolism can also influence the efficacy of drug combinations. Drugs in a combination can enhance or interfere with other drugs’ actions, leading to synergistic and antagonistic interactions [8]. For example, combinations involving bacteriostatic antibiotics, which inhibit cell growth, and bactericidal antibiotics, which induce cell death, are typically avoided in the clinic due to their antagonistic interaction [9]. This antagonism is hypothesized to occur due to their opposing effect on cellular respiration [10]. While recent studies have focused on the influence of metabolic environment (i.e. availability of nutrients, oxygen, extracellular metabolites) on individual drugs, a systematic analysis of the impact of metabolic environment on drug interactions is lacking. It is unclear if drug interactions are sensitive or robust to the environment.

Understanding the impact of metabolic environments on antibiotic efficacy is essential for clinical translation of antibiotic therapies discovered from in vitro screens [11]. This can ultimately help predict the impact of gut and tissue microenvironment on antibiotic susceptibility. Further, knowledge of the pathogen metabolic environment is critical for treating slow-growing pathogens like *M*. *tuberculosis* and targeting pathogen biofilms.

Yet traditional *in vitro* testing is typically done in a single metabolic condition [11]. Given the large space of possible metabolic environments *in vivo* [12], *in silico* algorithms are needed to predict the impact of various metabolic environments on drug combinations. Existing approaches to infer drug-drug interactions in both microbes and cancer cells, including the *INferring Drug Interactions using chemo-Genomics and Orthology* (INDIGO) approach that we previously developed [13], assume interaction outcomes are fixed for a drug combination.

To address this challenge of predicting the impact of metabolic environment on drug combination efficacy, here we develop the Metabolism And GENomics-based Tailoring of Antibiotic regimens (MAGENTA) approach. This systems-biology approach comprehensively captures the cellular processes involved in drug action and drug interactions such as stress response, drug transport and metabolism. MAGENTA achieves this by harnessing chemogenomic profiles associated with both distinct metabolic environments and drugs. Chemogenomic screens measure fitness of gene knockout strains treated with drugs and stress agents of interest [14]. MAGENTA identifies genes that significantly impact fitness when exposed to drugs or metabolic stressors from chemogenomics data resulting in a set of drug-gene and metabolic environment-gene interactions. It then uses these chemical-genetic interactions to predict drug-drug interactions in a new environment. MAGENTA applies a machine learning algorithm called Random Forests to identify a core group of genes in the chemogenomic profiles that are predictive of drug synergy and antagonism across metabolic environments.

MAGENTA enables, for the first time, the prediction of impact of metabolic conditions on antibiotic combination efficacy. We then experimentally validate this approach by testing predictions involving several drug combinations across distinct metabolic conditions in *E*. *coli*. In addition, we apply the *E*. *coli* MAGENTA model to predict interactions in the pathogen—*Acinetobacter baumannii*, by identifying genes that are conserved between the two species. *A*. *baumannii* is frequently associated with multi-drug resistance and is ranked as one of the most dangerous pathogens in hospitals worldwide by the Infectious Diseases Society of America [15]. Here we identified drug combinations that are synergistic across multiple conditions in *A*. *baumannii* by overlaying orthologous genes on to the *E*. *coli* MAGENTA model. This orthology mapping approach can enable the application of data from expensive chemogenomics and drug-interaction screens in model organisms for identifying synergistic combinations in several related bacterial pathogens with genome sequence information.

## Results

### The metabolic environment significantly impacts drug combination efficacy

**Fig 1:**
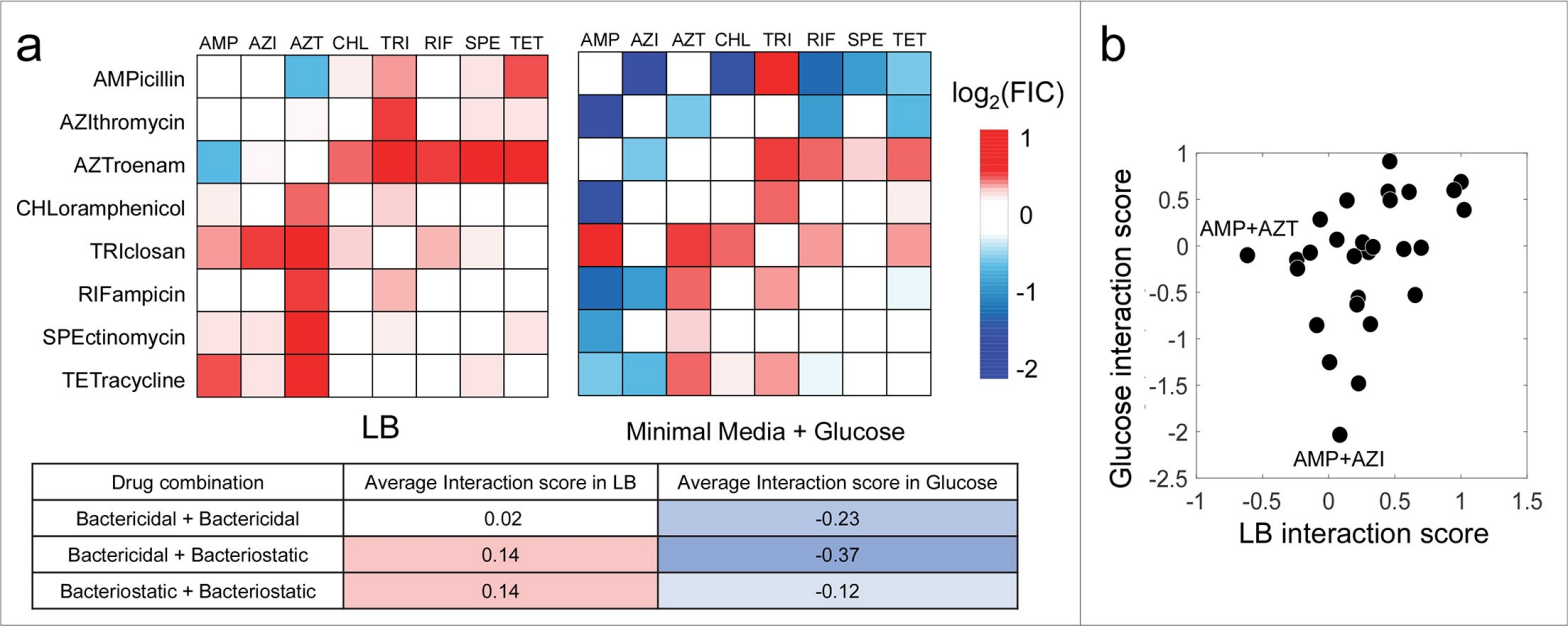
Antibiotic interactions change significantly in different growth environments. (a) Heat maps represent all pairwise interactions among 8 antibiotics in rich media (LB) and minimal media supplemented with glucose. Blue, white or red boxes correspond to synergistic, additive or antagonistic pairs. All drug combinations on average showed a significant shift towards synergy in glucose media. This was strongly pronounced for combinations involving both bactericidal and bacteriostatic drugs. The average interaction score for each class of drug combinations is shown in the table. (b) Scatter plot comparison of interaction scores in rich media versus glucose minimal media. The log transformed FIC interaction score is shown. While there is a significant correlation between interaction scores in the different media conditions, there are salient differences. Outlier combinations that have divergent outcomes in each condition are highlighted. For example, ampicillin is synergistic with azithromycin only in minimal media.

To understand the impact of metabolic environment on drug interactions, we experimentally measured all pairwise interaction outcomes of 8 antibiotics against *E*. *coli* cells grown in LB (rich) and M9 glucose (minimal) media (Fig 1). We used the popular Loewe-additivity model and the Fractional Inhibitory Concentration (FIC) metric to quantify the drug interactions [16,17] (Methods). The FIC for a drug combination is obtained by adding the fractional MIC (i.e. Minimum Inhibitory Concentration) of each drug in a combination. The FIC scores were log2-transformed for ease of interpretation. Negative log-FIC scores (i.e. < 0) imply synergy, i.e. the same amount of growth inhibition is achieved with a lower dose when both the drugs are combined. Antagonistic interactions have a positive log-FIC score.

Analysis of our experimental drug interaction FIC scores revealed that change in metabolic state strongly influenced sensitivity to drug combinations. The drug interaction FIC scores changed considerably between the two conditions, with only 42% of the combinations showing the same direction of interaction (i.e. antagonism–log-FIC > 0.2, synergy–log-FIC < -0.2). Interestingly, interactions were significantly more synergistic in glucose media (mean log-FIC = -0.13) compared to LB (mean log-FIC = +0.28; p-value = 0.001, paired t-test). Interactions involving combinations of bactericidal and bacteriostatic drugs showed the strongest difference between the two conditions compared to other drug combinations (Fig 1). These combinations became strongly synergistic in glucose media from being weakly antagonistic in LB media (mean log-FIC = -0.37 in glucose media, mean log-FIC = +0.14 in LB; p-value = 0.08, paired t-test).

These results suggest that drug interaction outcomes are not fixed for a drug combination and can change significantly depending on the metabolic environment. This further complicates the challenge of predicting drug interactions–in addition to the large space of drugs and dosage, the metabolic environment of the pathogen should also be considered.

### Predicting drug–drug interactions in diverse metabolic environments using MAGENTA

To account for this variability in drug interactions, we developed the MAGENTA framework to predict the impact of metabolic environment. MAGENTA takes as input the chemogenomics data of individual drugs and known drug-drug interaction training data, and outputs the predicted interaction score for a list of novel drug combinations (Methods). To predict the impact of metabolic state on drug sensitivity, we first identify genes that impact fitness during growth in distinct metabolic conditions from chemogenomic profiling. We then model metabolic perturbations using a similar framework for modeling drugs, and subsequently predict the impact of metabolic state on drug combination efficacy.

We introduce two key features in MAGENTA to enable prediction of metabolic impact on drug sensitivity, which is not possible using existing drug interaction tools such as INDIGO or *Overlap2 Method* (O2M) [18]. Firstly, MAGENTA uses chemogenomic profile of the metabolic condition as input, in addition to using chemogenomic profiles of the drugs in a combination. This involves the integration of three different chemogenomic profiles. While existing chemogenomics frameworks can predict interactions between pairwise combination of drugs, here we show that MAGENTA can make predictions of combinations of multiple stress agents (i.e. > 2). This allowed us to simulate the impact of metabolic conditions on drug interactions. The mathematical framework used by MAGENTA for quantifying drug chemogenomic profile similarity and uniqueness respectively are directly scalable to multiple combinations (S1 Fig). To account for dosage, we re-scale the scores by normalizing them by the number of drugs in a combination in order to achieve the same units as the model defined for two drugs.

Further, we found that chemogenomic profiles of metabolic conditions differ distinctly from chemogenomic profiles of drugs and other stress agents (S2 Fig). This is because, while drugs have significant number of chemical-genetic interaction with genes that confer both resistance and sensitivity, nutrients such as glucose predominantly contain genes that confer sensitivity. Hence, as a second addition to the MAGENTA framework, we also used the genes that confer resistance as input. Data on genes conferring resistance is relevant here for differentiating media conditions from drugs.

### Experimental validation of model predictions for 56 three-way drug combinations

We trained MAGENTA using our experimental pairwise drug-drug interaction data in LB and glucose media (Fig 1) along with data for 171 drug pairs generated in our prior study [13]. We used chemogenomic profiles for these drugs and metabolic perturbations from the Nichols et al. study [19], which screened 3979 gene-deletion strains of *E*. *coli* with 72 drugs and 8 metabolic perturbations.

**Fig 2:**
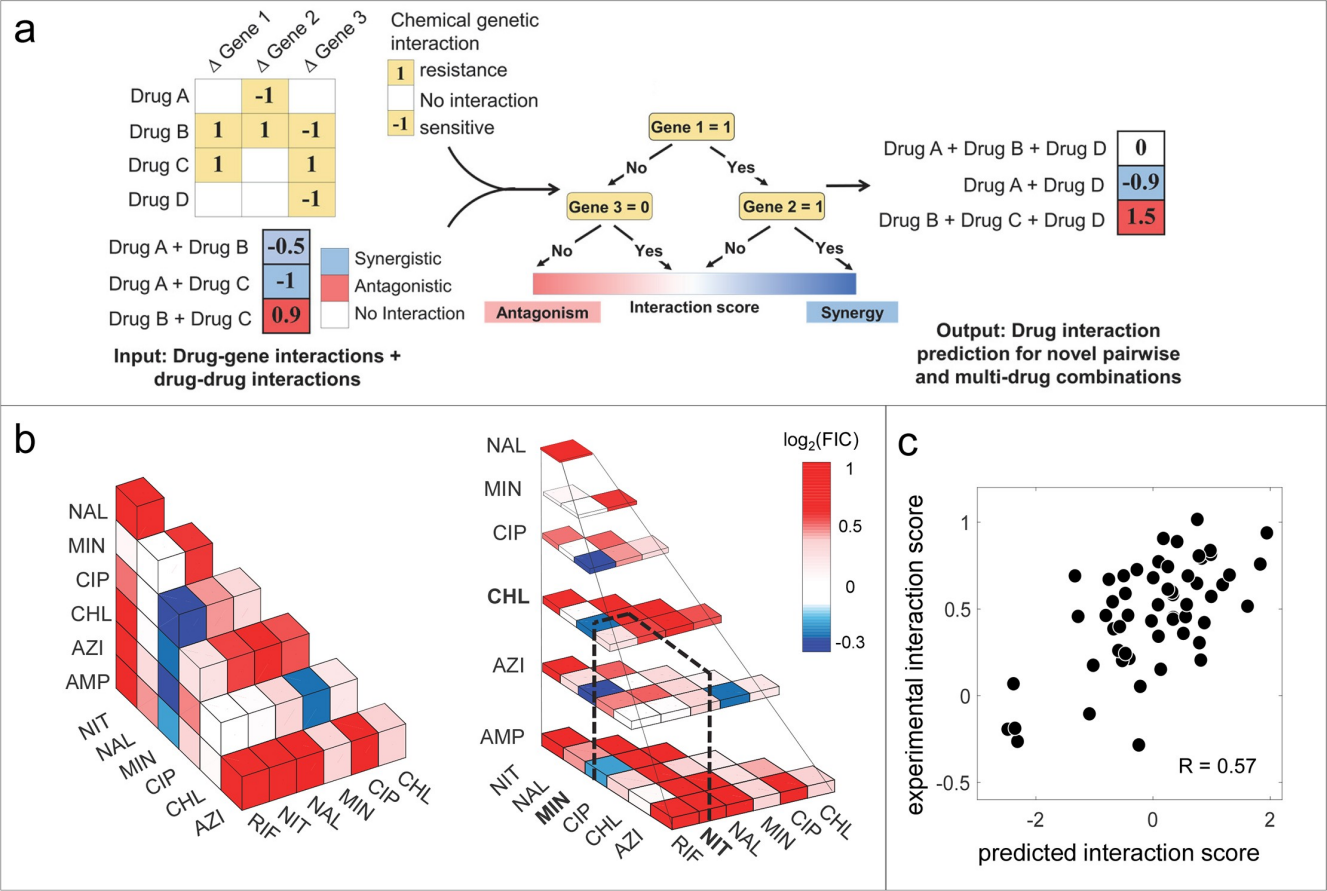
Predicting multi-drug combinations using MAGENTA. (a) Schematic workflow of MAGENTA approach. MAGENTA takes drug chemogenomic profiles and interactions among drugs as input. This is used to train a Random Forest Model which can predict synergy and antagonism among pair-wise or higher-order combinations of new drugs given their chemogenomic profiles. (b) All three-way interactions among 8 antibiotics are represented as 3D heat-map. Blue, white or red boxes correspond to synergistic, additive or antagonistic three-way combinations. Among 56 combinations, only Azi+Min+Rif and Min+Cip+Rif exhibit strong synergy. The three-way interactions among 8 antibiotics are also represented as layers for maximum visibility. The dotted lines depict the interaction between nitrofurantoin, minocycline and chloramphenicol. (c) Scatter plot comparison of MAGENTA 3-way interaction predictions and experimental measurements demonstrate that MAGENTA can robustly identify 3-way antibiotic synergy and antagonism (rank correlation R = 0.57, p = 5 x10^-6^).

Since the MAGENTA framework integrates multiple chemogenomic profiles, we first confirmed MAGENTA’s ability to predict the outcome of multi-drug combinations from individual drug chemogenomic profiles. To test the predictions by MAGENTA, we experimentally measured 56 three-way combinations involving 8 antibiotics in LB media. These 8 antibiotics included drugs with distinct targets and mechanism of action (Fig 2; Table 1). Comparison of MAGENTA predictions with experimental measurement revealed that it accurately predicted three-way drug interaction outcomes with significant correlation (Rank correlation R = 0.57, p-value = 5 x 10^−6^; Fig 2). We observed a similar accuracy for predicting pairwise drug combinations in our prior study [13]. This suggests that the MAGENTA framework can be seamlessly extended to multi-drug combinations. Further, we observed that the majority of the three-way interaction outcomes were surprisingly antagonistic and only 2 out of the 56 combinations showed synergy (interaction score < -0.2). Given the uneven distribution of synergy and antagonism, we also assessed MAGENTA predictions using the Anova statistic and found that the predicted interaction scores differed significantly for synergy and antagonism respectively (p-value = 0.0003; S3 Fig). The high predominance of antagonism underscores the need for a computational approach to discover synergistic combinations.

**Table 1:** List of drugs used in this study, their MIC, activity (bactericidal (C), bacteriostatic (S), or both (CS) in *E*. *coli*) and their targets are shown. Drug annotations are from the Nichols et al study.

			E. coli (MIC μg/ml)			A. baumannii (MIC μg/ml)		
Antibiotic	Abbreviation	Target	LB	Glucose	Glycerol	LB	Glucose	Glycerol
Amikacin (C)	Amk	Ribosome (30S)			2.8	14	16	25
Ampicillin (C)	Amp	Membrane	14	8	14	28	40	40
Azithromycin (S)	Azi	Ribosome (50S)	2	16	6			
Aztroenam (C)	Azt	Membrane	0.03	46	20	8	12	22
Cefoxitin (C)	Cef	Membrane			3.6			
Chloramphenicol (S)	Chl	Ribosome (50S)	2.4	6.6	4			
Ciprofloxacin (C)	Cip	DNA gyrase	0.01					
Minocycline (S)	Min	Ribosome	4					
Nalidixic acid (C)	Nal	DNA gyrase	28		50	8	9	8
Nitrofurantoin (CS)	Nit	Multiple mechanisms	10					
Rifampicin (C)	Rif	RNA Polymerase	7	14	20	0.7	0.2	1.2
Spectinomycin (S)	Spe	Ribosome (30S)	10	60	40			
Tetracycline (S)	Tet	Ribosome (30S)	0.9	1.6	1.4	0.4	0.8	0.7
Triclosan (CS)	Tri	Membrane; fatty acid synthesis	1.4	1.8	1.8			

### MAGENTA accurately predicts drug–drug interactions in a novel metabolic environment

Having confirmed that MAGENTA can accurately predict three-way interactions, we then applied MAGENTA to predict the impact of metabolic environment on drug interactions. Out of the 8 metabolic conditions for which chemogenomic data was available from the Nichols et al study [19], we used MAGENTA to predict interactions in minimal media with glycerol as carbon source. We chose glycerol condition as *E*. *coli* is predicted to use a different metabolic state than glucose [20]. We hypothesized that this major shift in metabolism will have a significant impact on drug combination efficacy.

To validate the model predictions, we experimentally measured all 55 pairwise drug interactions of 11 antibiotics in glycerol media, in duplicate. In addition to the 28 drug combinations previously tested, we also tested 27 new drug combinations involving three new antibiotics that were not part of the glucose media training data. These three antibiotics—cefoxitin, nalidixic acid and spectinomycin, use distinct mechanisms of action compared to those drugs used in the training data set. Hence this validation data set would test the limits of the algorithm with interactions involving both new metabolic conditions and new drugs with distinct mechanism of action.

**Fig 3:**
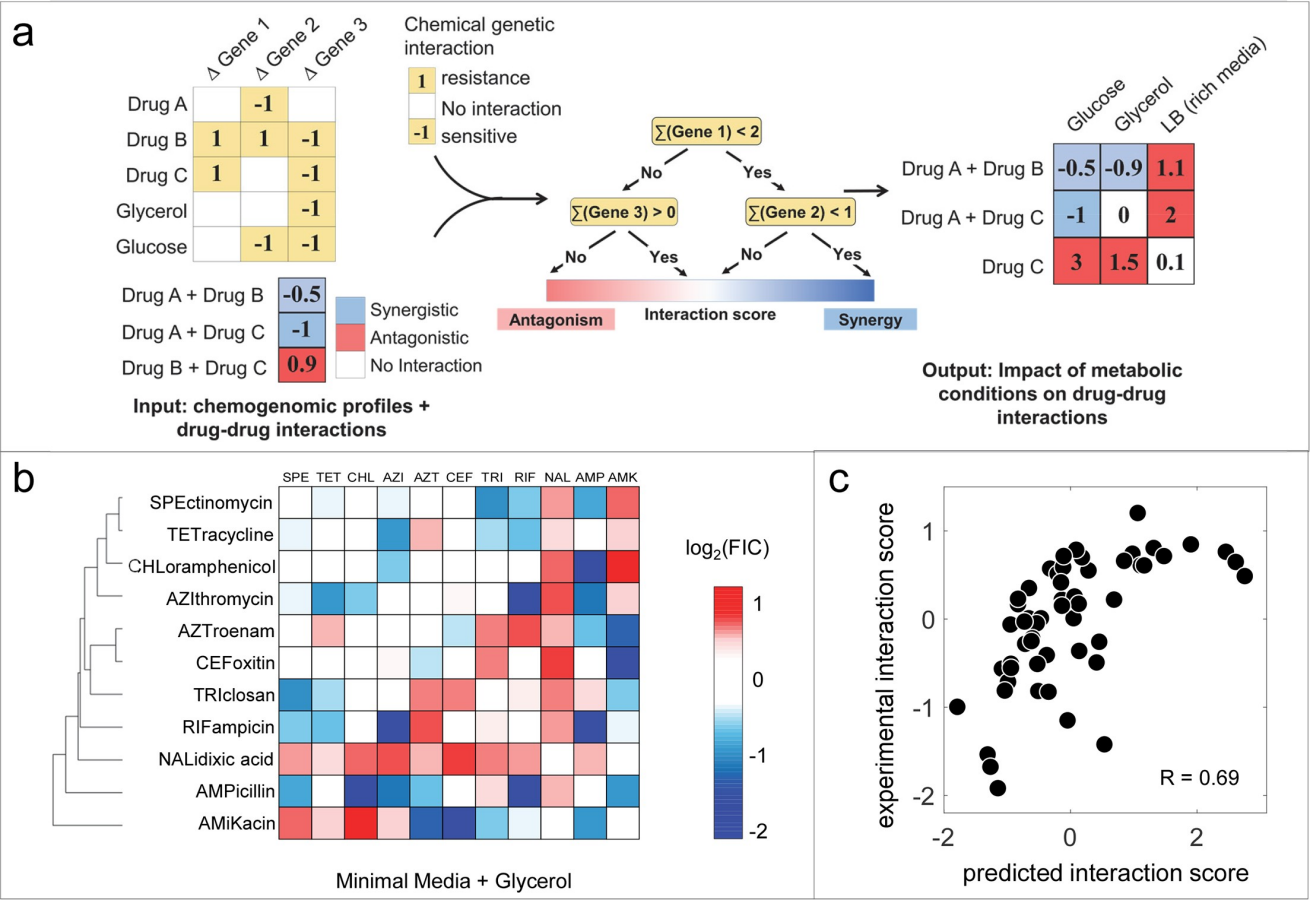
Predicting impact of novel metabolic environments on drug interactions using MAGENTA. (a) Schematic workflow of MAGENTA approach. MAGENTA takes drug chemogenomic profiles and interactions among drugs or between drugs and metabolic conditions as input. This is used to train a Random Forest model which can predict synergy and antagonism among pair-wise or higher-order combinations of new drugs in novel metabolic conditions given their chemogenomic profiles. (b) All pairwise interactions among 11 antibiotics in minimal media supplemented with glycerol are represented as a heat map. Blue, white or red boxes correspond to synergistic, additive or antagonistic combinations. Clustering of interaction scores was done using Euclidean distance and average linkage (Unweighted average distance (UPGMA)). (c) Scatter plot comparison of MAGENTA predictions and experimental measurements in glycerol minimal media demonstrate that MAGENTA can robustly predict antibiotic synergy and antagonism in new environments (rank correlation R = 0.69, p = 1x10^-8^).

In contrast to the glucose condition, MAGENTA did not predict strong synergy between bacteriostatic and bactericidal drugs in glycerol media. For example, MAGENTA predicted that combination of ampicillin, a bactericidal drug, and tetracycline, a bacteriostatic drug, were additive in glycerol media. Similarly, combination of aztreonam and azithromycin was also predicted to be additive in glycerol media. Experimental measurement of 55 drug combinations in glycerol media validated the MAGENTA predictions. Overall, comparison with experimental data revealed that MAGENTA accurately predicted interaction outcomes across all 55 drug combinations (Rank correlation R = 0.69, p-value = 1 x 10^−8^; Fig 3). Thus, the interaction scores predicted by MAGENTA accurately represent whether the metabolic perturbation can enhance or impede the efficacy of a drug combination.

Notably, a large subset of the test set involved new drug combinations for which we do not have training data. For the drug combination subset that was shared across all conditions (28 pairs), the overall consistency between conditions was lower than the predictions from MAGENTA. We split the interaction data as synergy (Interaction score < -0.2), neutral or antagonistic (> 0.2). The consistency was 32% (p-value = 0.5) for LB and glycerol, 53% (p-value– 6 x 10^−4^) for glucose and glycerol, 42% (p-value = 0.03) for LB and glucose. The consistency for MAGENTA was 65% (p-value = 1 x 10^−6^) for this subset and 62% overall for 55 combinations (p-value = 4 x 10^−8^). P-values were estimated by comparison of observed consistency with 1,000 random permutations from the training data set using a t-test.

The extent of change in interaction outcomes in glycerol media was influenced by the bacteriostatic and bactericidal nature of the drugs in the combination. Interactions involving combinations of bactericidal and bacteriostatic drugs did not show strong synergy in glycerol growth condition (mean log-FIC = -0.04), in contrast to our observation in glucose minimal media (mean log-FIC = -0.37). Surprisingly combinations involving two bacteriostatic drugs showed the strongest synergy (mean log-FIC = -0.28). Hence while the correlation was high between conditions for the subset of shared combinations, (R = 0.54 between LB and glucose, 0.64 between LB glycerol, and 0.77 for glucose and LB), it doesn’t represent the shift towards synergy or antagonism. The mean of the glucose interaction scores was -0.13, while for glycerol it was -0.27 for the combinations that overlapped and +0.26 for those that didn’t (S6 Fig). MAGENTA had a similarly high correlation of 0.78 for this subset, but also captured both the shift toward synergy and the relative ordering (S7 Fig, S8 Fig). To make predictions for a new condition using MAGENTA, instead of measuring all pairwise combinations of n drugs (n choose 2) across different media, only n chemogenomic profiles are needed along with a small training data set.

Growth in glycerol had a unique impact on drug interactions compared to LB and glucose media. Drugs that depend on facilitated transport like the aminoglycoside antibiotics—spectinomycin and amikacin, were more synergistic in glycerol media, possibly due to increased active uptake due to the higher activity of TCA cycle in glycerol condition [21]. The difference in interaction outcomes might also occur due to osmotic stress induced by glucose [22]. Drugs such as ampicillin, aztreonam and triclosan that disrupt bacterial cell wall were more synergistic in glucose media. The synergistic effect of ampicillin and triclosan is hypothesized to be due to the disruption of the cell wall by these drugs resulting in enhanced cellular penetration of a second drug [23]. The osmotic stress in glucose media relative to other conditions will further enhance the synergy of cell wall disrupting drugs by increasing membrane permeability. The differences in drug interaction outcome in glycerol media highlight the impact of metabolic environment on drug combinations.

The drug interaction validation data in glycerol media was used as additional training data for MAGENTA to further improve its accuracy. The accuracy of the updated MAGENTA model was then assessed through ten-fold cross validation analysis. Through cross validation analysis we found that even higher accuracies (Rank correlation R = 0.7; Methods) can be obtained if some training data involving the corresponding condition or drug is provided as input for MAGENTA (S5 Fig). We use this final MAGENTA model for predicting drug interactions in different environments and to identify genes predictive of drug interactions.

### Metabolic pathways predictive of drug synergy and antagonism

While the chemogenomic profiling data used as input to MAGENTA encompasses 3979 genes, analysis of MAGENTA model revealed that a small subset of genes was sufficient to explain most of the model’s predictive ability. The top 60, 319 and 867 genes are sufficient to predict 50, 75, and 95% of the model’s predictive ability.

Several metabolic pathways were over-represented among the top predictive genes (Table 2; S1 Table). Genes in the oxidative phosphorylation pathway showed the highest extent of over-representation among the top predictive genes (Table 2). The very high enrichment of the oxidative phosphorylation pathway relative to other cellular processes is consistent with the fact that this pathway is related to both drug sensitivity, drug-drug interactions and drug-media interaction [10,24,25]. In addition to this pathway, other top pathways were related to target processes of antibiotics like cell wall synthesis, DNA recombination and DNA mis-match repair. In addition, pathways related to drug transport and resistance were also over-represented among the top predictive genes. Analysis of top predictive genes for making three-way drug interaction predictions also revealed a significant enrichment for metabolic genes (S2 Table, S3 Table). Notably, the gene glmS, which was identified as the most predictive gene in our INDIGO model, was among the top ten predictors in the MAGENTA model as well for predicting three-way interactions and interactions across media conditions. glmS is a metabolic enzyme that catalyzes the first step in hexosamine pathway which produces precursors for cell wall synthesis and biofilm formation.

**Table 2:** Enriched biological pathways among the top predictive genes in the MAGENTA model. Pathway annotations are from the KEGG database. The table also shows the top pathways associated with drug synergy and antagonism. The presence of genes associated with these pathways in the drug chemogenomic profile was associated with synergistic or antagonistic interaction outcome.

Top Enriched Pathways	p-value
Oxidative phosphorylation	2.10E-06
Alanine, aspartate and glutamate metabolism	0.000121
Purine metabolism	0.000303
Phenylalanine, tyrosine and tryptophan biosynthesis	0.000656
Mismatch repair	0.000716
Lipopolysaccharide biosynthesis	0.001069
Homologous recombination	0.001541
Glycine, serine and threonine metabolism	0.001598
**Top Pathways (Synergy)**	p-value
Glycolysis / Gluconeogenesis	0.001284
Phenylalanine, tyrosine and tryptophan biosynthesis	0.001383
Selenocompound metabolism	0.005778
Citrate cycle (TCA cycle)	0.017201
**Top Pathways (Antagonism)**	p-value
Porphyrin and chlorophyll metabolism	0.005778
Galactose metabolism	0.008133
Glyoxylate and dicarboxylate metabolism	0.012333

The interaction outcomes for each drug combination in a metabolic condition depends on complex interplay between many genes. Nevertheless, the presence of genes associated with specific pathways in the chemogenomic profiles of the drugs or metabolic conditions can be a strong predictor of synergy or antagonism. When the chemical-genetic interaction with the genes in these pathways change in a new condition, it influences MAGENTA predictions of drug synergy. For example, we found that presence of genes in the glycolysis and TCA cycle pathway in the drug chemogenomic profile were strongly associated with synergy (p-value = 0.001 and 0.01 for glycolysis and TCA cycle respectively, hypergeometric test). Surprisingly, we found that genes in the galactose and glyoxylate metabolism pathway were the top predictors of antagonism (p-value = 0.008 and 0.01 respectively, hypergeometric test; Table 2). Increased activity of the glyoxylate pathway has been previously found to reduce sensitivity to antibiotics in both *M*. *tuberculosis* and *P*. *aeruginosa* by decreasing the activity of the TCA cycle [6,7]. Our results suggest that drugs or metabolic conditions that increase the activity of the glyoxylate pathway may result in antagonistic interactions. The presence of the glyoxylate- and TCA cycle- pathways on opposing sides of the drug interaction outcomes supports the validity of MAGENTA in inferring the underlying mechanisms influencing drug interaction outcomes.

To further understand the underlying mechanism behind the differences in interaction outcomes between the media conditions, we compared their corresponding chemogenomic profiles and identified genes unique to each condition. If the chemical genetic interactions of the top predictive genes change significantly in a new condition, it impacts the predicted interaction scores by MAGENTA. The gene sensitivity profile in glucose condition contained significantly higher number of genes encoding transporters, two-component sensors, efflux pumps (drug resistance genes) and sugar metabolism enzymes compared to glycerol condition (S4 Table). The predominance of transporters is consistent with the fact that glucose requires active transport while glycerol can enter the cell by passive diffusion through the membrane. The differential use of transporters and efflux pumps may further alter sensitivity to drugs that require active uptake.

### Predicting drug interactions across metabolic environments in new bacterial species using orthology mapping

Since drug interactions changes across metabolic conditions depended on pathways such as glycolysis that are highly conserved across evolution, we next tested the conservation of metabolism-related drug interaction changes in clinically-relevant organisms. In our prior study we discovered that the extent of conservation of drug interaction related genes identified by INDIGO were predictive of drug-drug interaction conservation between species. This enabled us to use widely available chemogenomic data in *E*. *coli* to make predictions for pathogens such as *S*. *aureus* and *M*. *tuberculosis* that are difficult to study and lack chemogenomics data. In this study, we tested the conservation of metabolism-related drug interaction changes in the pathogen *A*. *baumannii*.

Due to rising resistance, drug combination therapy is being explored for treating *A*. *baumannii* infections [26]. *A*. *baumannii* is an opportunistic pathogen causing pneumonia, skin-, wound, urinary-tract, brain- and bloodstream infections [27]. The metabolic flexibility of *A*. *baumannii* is predicted to contribute to its persistence and colonization [27,28]. Hence, given the importance of drug combinations to treat this pathogen and its metabolic flexibility, we focused on the impact of pathogen metabolic environment on drug interactions in *A*. *baumannii*.

Genes that were orthologous between *E*. *coli* and *A*. *baumannii* were obtained from OrtholugeDB and mapped onto the MAGENTA model [29]. Overall, we found 1180 genes in *A*. *baumannii* that were orthologous with genes in the *E*. *coli* MAGENTA model. These orthologous genes were highly enriched among the top 319 drug interaction predictive genes (p-value = 7 x 10^−5^, hypergeometric test). Among the top predictive genes, pathways in central metabolism were conserved between the two species, while genes in lipopolysaccharide synthesis & DNA mis-match repair were not conserved (S5 Table). Thus, we hypothesized that many of the drug interaction outcomes across metabolic conditions will be conserved between the two species.

Using this MAGENTA *A*. *baumannii* model, we predicted all 15 pairwise interaction outcomes of 6 antibiotics in three media conditions we previously studied using *E*. *coli*–LB, glucose minimal and glycerol minimal media. These 6 drugs were chosen based on their efficacy in both *E*. *coli* and *A*. *baumannii*, and the availability of chemogenomics data. In addition, two drugs–amikacin and nalidixic acid, were included as they were not measured in the glucose media training data for MAGENTA. The 45 drug interactions across three media conditions, including 13 novel interactions involving the two new drugs will allow us to assess the accuracy of MAGENTA for predicting interactions in a new organism involving drug-media combinations that it was not trained on.

**Fig 4:**
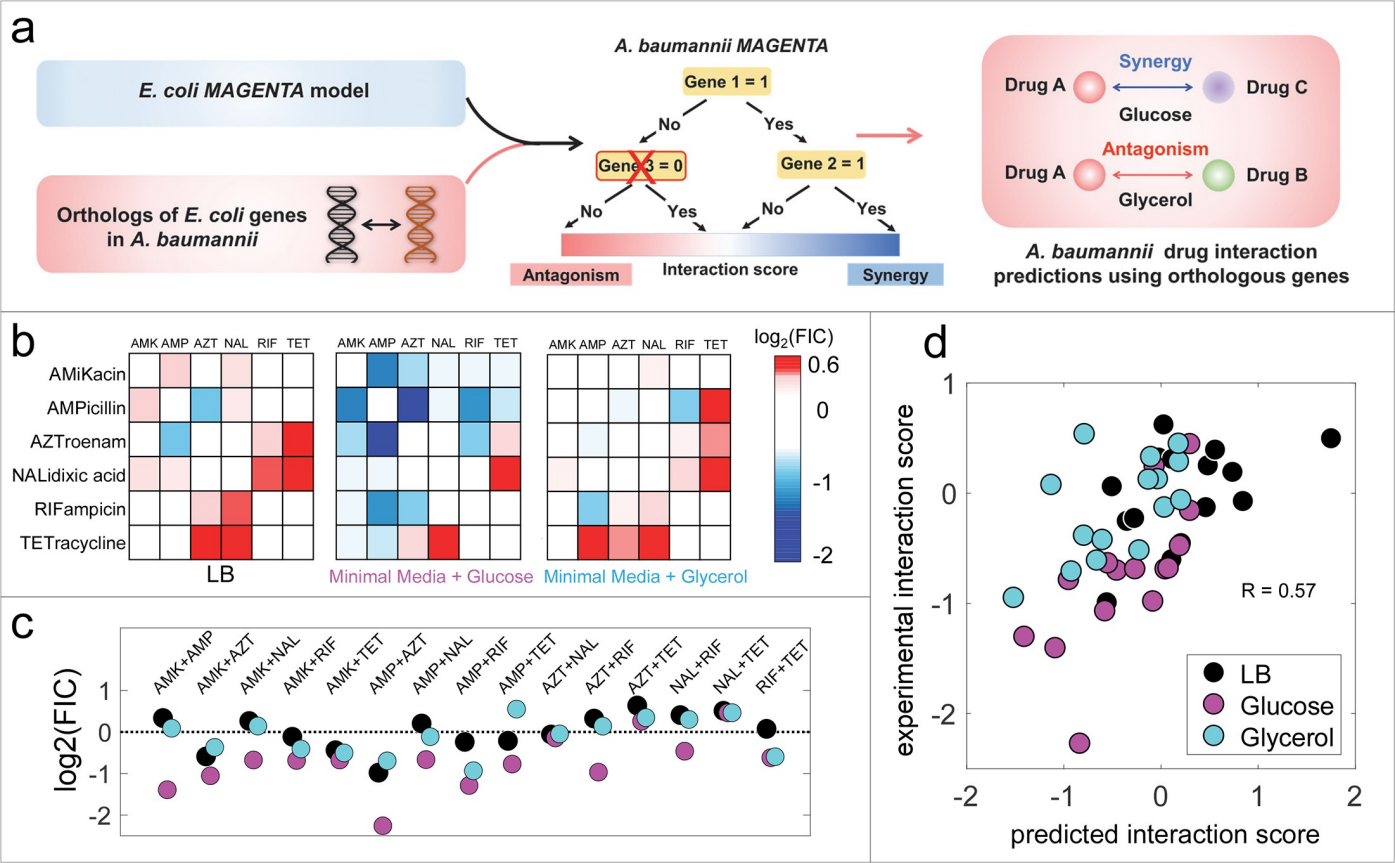
Predicting the impact of metabolic environments on drug interactions in *A*. *baumannii* using MAGENTA. (a) Schematic workflow of the approach for predicting interactions in a new bacterial strain using *E*. *coli* drug interaction and chemogenomics data. Genes that are common between *E*. *coli* and *A*. *baumannii* are overlaid onto the *E*. *coli* MAGENTA model. The non-orthologous genes were deleted (i.e. they were set to be zero) and interaction outcomes were predicted using the conserved orthologous genes alone. (b) All pairwise interactions among 6 antibiotics in three media conditions for *A*. *baumannii* are shown as heat maps. Blue, white or red boxes correspond to synergistic, additive or antagonistic combinations. (c) Comparison of the interaction scores for each drug combination in three media conditions identified combinations that are sensitive to the environment. For example, ampicillin-tetracycline combination is synergistic, additive and antagonistic in glucose, LB and glycerol environment, respectively. (d) Scatter plot comparison of media specific interaction scores predicted by MAGENTA and experimental measurements demonstrate that MAGENTA can robustly predict antibiotic synergy and antagonism in various environments for a new species using *E*. *coli* data (Rank correlation R = 0.57, p = 5x10^-5^).

Overall, the predicted drug-drug interaction scores by MAGENTA across the three media conditions significantly correlated with the measured interaction scores (Rank correlation R = 0.57, p-value = 0.0001; Fig 4). The interaction scores for *E*. *coli* and *A*. *baumannii* showed significant correlation for the 32 drug interactions that were experimentally measured in both species (Rank correlation R = 0.57, p-value = 5 x 10^−5^). This suggests that drug interactions are relatively conserved between the two species and is consistent with our observation that top drug interaction predictive genes in MAGENTA were significantly conserved between the two species. However, even for cases where there is no *E*. *coli* interaction data available in that specific metabolic condition, MAGENTA can accurately predict interaction outcomes with equally high correlation (R = 0.57) as demonstrated here using the drugs amikacin and nalidixic acid.

While majority of antibiotic combinations that were synergistic in *E*. *coli* were also synergistic in *A*. *baumannii*, combinations of amikacin and tetracyline, showed synergy in *A*. *baumannii* but were antagonistic in *E*. *coli* in LB media. In contrast, combination of amikacin and ampicillin were synergistic in *E*. *coli* in glycerol media but not in *A*. *baumannii*. Importantly, MAGENTA correctly identified combinations that differed between the two species. The predicted extent of difference in interaction outcome correlated significantly with the observed extent of interaction change (rank correlation R = 0.59, p-value = 2 x 10^−5^).

We also observed significant difference in interaction outcome for ampicillin and aztreonam, which target cell wall synthesis. For instance, combination of aztreonam with ampicillin was synergistic in *A*. *baumannii* but antagonistic in *E*. *coli* in glucose media. This observation is in agreement with the lack of conservation of the cell wall lipopolysaccharide (LPS) synthesis pathway between the two species. While LPS synthesis is essential in *E*. *coli*, it is dispensable in *A*. *baumannii* [30].

### Landscape of 2556 drug interactions in nine distinct metabolic environments reveals robust synergistic combinations

**Fig 5:**
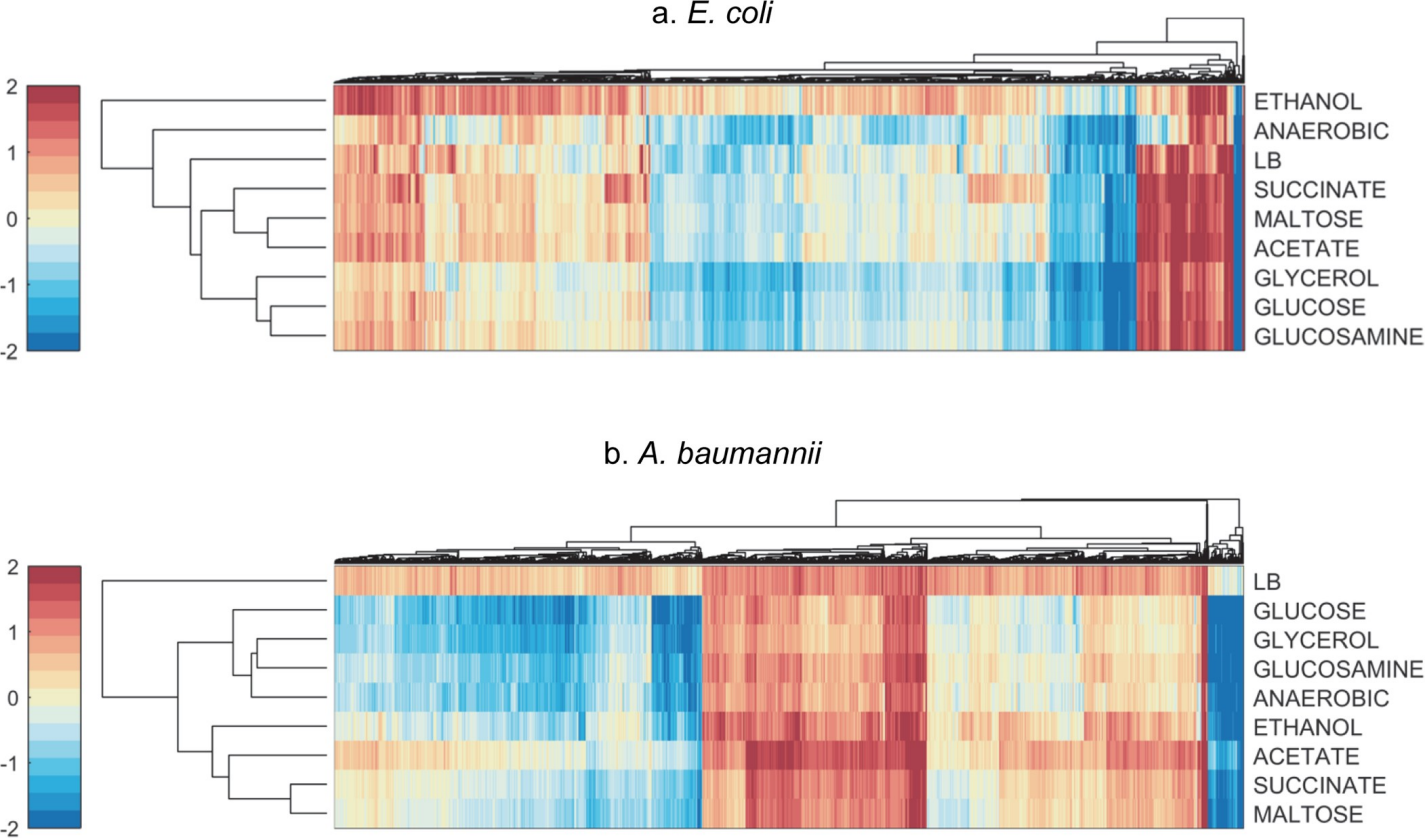
The drug-drug interaction landscape. (a) The heat maps show the predicted impact of 9 distinct metabolic environments on the interaction outcomes of 2556 pairwise drug combinations (synergy (blue), antagonism (red)). The drug combinations and metabolic conditions are clustered based on similarity (Euclidean distance) and the dendrogram was plotted based on average linkage. Panel b shows the corresponding interaction scores for *A*. *baumannii*.

Some drug combinations, such as amikacin-tetracycline and ampicillin-aztreonam showed robust synergy across all three conditions in *A*. *baumannii*. These combinations might serve as promising leads for treating *A*. *baumannii*. To discover other combinations in both *A*. *baumannii* and *E*. *coli* with broad spectrum metabolic synergy, we predicted interaction outcomes for 2556 pairwise drug combinations involving 72 drugs across 9 growth conditions for which we had chemogenomics data (Fig 5). These 9 metabolic conditions—namely growth in glucose, glucosamine, glycerol, acetate, maltose, succinate, ethanol minimal media, aerobic and anaerobic growth in LB, represent a wide spectrum of potential metabolic states for the bacteria. *In vivo* metabolic conditions span growth in diverse substrates such as sugars, nucleotides, glycerol, lipids and hypoxic conditions [12] and these 9 metabolic conditions studied here are representative of the conditions *in vivo*.

The nine metabolic conditions uniquely impacted drug interaction outcomes. Growth in anaerobic condition had a very strong and distinct impact on drug interactions compared to other conditions (Fig 5). Given that oxidative phosphorylation was the top predictive pathway for drug interactions across media conditions, growth conditions that change the activity of this pathway exert strong influence on interaction outcomes. In *E*. *coli*, we identified 119 combinations out of the 2556 combinations screened that were synergistic across all metabolic conditions. For example, we found that combinations of azithromycin and rifampicin, and ampicillin and chloramphenicol were synergistic across all 9 metabolic conditions (S6 Table). Several combinations used clinically also showed robust synergy across these conditions. The list of 119 combinations included combinations of rifampicin with tetracycline, ampicillin, azithromycin and clarithromycin. These combinations with rifampicin are frequently used for treating biofilm associated infections [31]. In addition to rifampicin, the antibiotics—ampicillin, vancomycin and fusidic-acid were also over-represented in the list of 119 combinations with robust synergy.

Analysis of drug interaction landscape in *A*. *baumannii* revealed 19 combinations that showed synergy across all the growth conditions (Interaction score < -0.5, S6 Table). This represents just 0.74% (1 out of 134) of the total combinations screened. Thus, MAGENTA can potentially reduce the search space by 100-fold compared to a trial and error approach or a blind screen.

The accurate estimation of drug interactions in *A*. *baumannii* was possible because of the conservation of top predictive genes in the *E*. *coli* MAGENTA model in *A*. *baumannii*. To assess the generalizability of this approach, we compared the conservation of the top predictive genes in the pathogens *S*. *aureus* and *M*. *tuberculosis*. Top genes predicted by MAGENTA were enriched for those that are conserved between the two species. We found a significant enrichment for orthologs of *S*. *aureus* (1 x 10^−5^) and *M*. *tuberculosis* (2 x 10^−8^). This suggests that we can apply MAGENTA model to make accurate predictions across metabolic environments in these systems as well.

We have predicted interaction outcomes for 2556 pairwise drug combinations involving 72 drugs across 9 growth conditions for these two organisms (S9 Fig). Analysis of drug interaction landscape in these two pathogens revealed 113 and 108 combinations that showed strong synergy across all the growth conditions in *M*. *tuberculosis* and *S*. *aureus* respectively (Interaction score < -0.5, S7 Table). Of note, we identified robust synergistic interactions involving the frontline drugs used for treating Tuberculosis. We found that the antibiotics azithromycin and fusidic acid had robust synergy with the Tuberculosis drugs rifampicin and isoniazid respectively. Overall, this dataset can be used to prioritize combinations effective in specific growth conditions and potentially used to identify metabolically-robust drug combinations.

## Discussion

In this study we found that the pathogen metabolic environment significantly modulates drug combination efficacy. This trend was observed across nine different metabolic conditions and several antibiotics spanning various target processes; each metabolic state had a distinct and unique impact on each drug. This observation greatly complicates the search for finding effective therapies, given the wide range of metabolic conditions that pathogens encounter *in vivo* or in biofilms. The differences in sensitivity between metabolic conditions may also explain the differences in efficacy *in vitro* and *in vivo* observed for drugs [11].

To address this challenge, in this study we developed a computational approach (MAGENTA) to predict how metabolic environments can impact drug combination efficacy. An important finding from this study is that interactions between drugs across metabolic conditions can be predicted based on chemogenomic profiles of the individual drugs and the metabolic perturbation. This suggests that metabolic stress can be modeled using a similar framework as drug induced stress. Our approach can be potentially extended to other stressors including antimicrobial proteases and toxins [32,33].

Multi-drug combinations can greatly reduce the rise of resistance and enhance potency compared to single agents [34]. However, the number of possible permutations increases exponentially with the number of drugs in a combination regimen; this greatly underscores the need for computational tools like MAGENTA to identify most synergistic combinations. Prior studies on predicting drug interactions using chemogenomics focus only on pairwise drug combinations; here we demonstrate that the MAGENTA approach accurately predicted interaction outcomes involving multi-drug combinations. Multi-drug interaction predictions are especially relevant for diseases like tuberculosis, where combinations of 4 antibiotics are commonly used for treatment.

Theoretical models suggest that pairwise combinations of underlying drugs can be used to predict triplet combinations [35,36]. To make predictions for all 3-way combinations of n drugs, only n chemogenomic profiles are needed along with a small training data set for MAGENTA, but (n choose 2) pairwise interactions are needed for the pairwise approach. MAGENTA is hence more effective in exploring large number of combinations. Furthermore, MAGENTA can make predictions for combinations with new drugs that it was not trained on using chemogenomics data. In our case, three out of the 8 tested drugs are not part of the training set. Out of the 56 triplet combinations, the pairwise approach can be used to make predictions for only 10 triplets using pairwise data in the training set.

Notably, MAGENTA was able to predict interaction outcomes in a new metabolic condition (glycerol) based on training data in glucose and LB media. This result corroborates the ability of MAGENTA to extract mechanistic features that influence drug interactions from chemogenomic profiles, such as the role of metabolic pathways. Our unbiased data-driven approach confirmed the importance of oxidative phosphorylation and cellular respiration pathway on antibiotic efficacy. Reducing respiration is known to inhibit bactericidal drug lethality [10]. The oxidative phosphorylation pathway likely affects antibiotic efficacy in multifarious ways including oxidative stress, redox homeostasis and facilitating drug import. Our analysis also revealed the opposing association of TCA cycle and glyoxylate pathway with drug synergy and antagonism respectively, supporting previous studies [6,7,10,37].

Interactions involving combinations of bactericidal and bacteriostatic drugs showed striking differences between different metabolic conditions. This is consistent with the sensitivity of this drug combination to cellular metabolic state [10]. While this combination is avoided in the clinic due to antagonism [9], our results suggest that this combination is not antagonistic during growth in glycerol and becomes strongly synergistic during growth in minimal glucose media. Antagonism between combinations of bactericidal and bacteriostatic drugs is predicted due to their opposing effects on cellular respiration and TCA cycle. Hence shifting *E*. *coli* to a growth condition with higher activity of TCA cycle will have a significant impact on respiration and efficacy of these drugs. This change in nutrient source will shift the balance in favor of one class of drugs over the other. Our data revealed that metabolic state not only influences combination involving both bacteriostatic and bactericidal drugs, as previously believed, but it also strongly influences combinations involving only bactericidal or bacteriostatic drugs.

The nine metabolic conditions studied here are representative of *in vivo* metabolic conditions such as the gut environment and biofilms. Our study takes the first step towards rational design of combination therapies that are robust to the *in vivo* environment. While we have focused on the impact of a single metabolic perturbation on antibiotics in this study, the *in vivo* environment is complex and dynamic. Future modeling efforts that expand the capability of MAGENTA to dynamic conditions can enable prediction of effective therapies.

Our analysis of the drug sensitivity landscape revealed that metabolic environments had a significant impact on the efficacy of drug combinations (Fig 5). Nevertheless, by searching through 2556 combinations, we identified a small subset that were synergistic across all metabolic conditions in both *E*. *coli* and *A*. *baumannii*. Such synergistic drug combinations are urgently needed for treating *A*. *baumannii* infections. This opportunistic pathogen is a frequent cause of drug resistant wound-, urinary tract- and pneumonia infections. It is responsible for 2–10% of all Gram-negative hospital infections [38]. *A*. *baumannii* infections display resistance to most antibiotics used in the clinic and new treatments are desperately needed [26].

A key limitation of our approach is the need for chemogenomic profiling data. However, with the development of single gene knockout libraries, chemogenomic profiling data is increasing in number. For instance, the Resistome database has a compendium of chemogenomic profiles for 230 different perturbations in *E*. *coli* [39]. Similar large compendiums exist for *S*. *cerevisae* [40]. This approach could hence be potentially applied to a wide range of drugs in both prokaryotic and eukaryotic systems. Furthermore, our theoretical framework can be extended for discovering effective anti-cancer drug combinations. Drug combinations are frequently used in cancer chemotherapy to reduce resistance [41–44]. Our approach can enable the identification of robust combination therapies targeting the tumor microenvironment.

While our validation data sets tested the algorithm’s predictive ability involving novel drugs and conditions, through cross validation we found that higher accuracies (Rank correlation R = 0.7) can be obtained if some training data is provided as input for the corresponding condition or drug. An optimal experimental design should involve sparse sampling of multiple drug combinations and metabolic conditions rather than exhaustive combinations of a few drugs in one condition. The use of defined media rather than complex undefined media containing yeast extract or serum can also greatly improve modeling efforts. Knowledge of in vivo metabolic environments can enable direct prediction of effective therapies.

In sum, our study demonstrates that metabolic environment can elicit significant effects on antibiotic combination efficacy. Our new approach MAGENTA goes beyond existing drug combination discovery platforms by predicting the impact of metabolic state on combination therapies. Further, we were able to leverage existing chemogenomic and drug interaction data in *E*. *coli* to infer antibiotic interactions across metabolic conditions in the pathogen *A*. *baumannii*. Our approach can enable identification of robust therapies tailored to the pathogen and the metabolic environment.

## Methods

### Experimental drug interaction assays

All drug interaction experiments were conducted using the diagonal method [36,45]. For each drug, we constructed a linearly increasing concentration range of 14 concentrations from 0 drug to a Minimum Inhibitory Concentration (MIC), which results in almost complete inhibition. For pairwise or 3-way interactions, we constructed a similar dose series, with the top concentration MIC/2 or MIC/3 of each constituent drug. For each pairwise drug interaction assay, sensitivity to linearly increasing doses (dose-response) were collected for two single drugs and a 1:1 mixture of two drugs. For each three-way drug interaction assay, dose-responses were collected for three single drugs and a 1:1:1 mixture of three drugs.

Cell growth in these two or three drug mixtures were compared to growth in single drug components to calculate FIC values. For this, we located the dose fraction that gives the same level of inhibition in the single drug or combination dose-responses. The dose that results in a defined inhibition level is divided by the “expected dose” that would give the same inhibition if the drugs in the combination were same drugs, resulting in the Loewe Additivity based FIC drug interaction measure. For example, when considering a 3-way interaction, if the 50%, 60% and 70% of the top dose of drugs A, B and C result in IC50, then the expected IC50 was defined as ~0.6. If the dose fraction of the combination that gives IC50 is 60%, this 3-way combination is additive. If it is smaller or larger than 0.6, it is synergistic or antagonistic, respectively. The FIC for a drug combination is obtained by adding the fractional MIC of each drug in a combination. The fractional MIC is calculated by dividing the dose of a drug when used in combination by the MIC of that drug when used individually. FIC is equal to 1 if drugs are additive, less or more than 1 if drugs are synergistic or antagonistic, respectively. In this study, we used the log2 of FIC values as drug combination interaction values. We have used a quantitative metric rather than discrete classification of interactions for two key reasons. Firstly, use of quantitative interaction scores allows for a quantitative validation of model predictions. Secondly, regression algorithms perform better with a continuous range of values.

For FIC calculation, we used the drug or mixture dose that resulted in 70% inhibition (IC70) throughout the analysis. This choice was guided by our initial analysis which showed this inhibition level results in the highest reproducibility among replicates (S10 Fig, S11 Fig, S12 Fig). We also show that the scores are robust to the IC choice, as scores obtained using different ICs in the range of IC60 and IC80 highly correlate (r > 0.8) (S11 Fig, S12 Fig).

*Escherichia coli* MG1655 and *Acinetobacter baumannii* Bouvet and Grimont ATCC 17978 were used as bacterial strains. All drugs were purchased from Sigma. MICs for each drug are provided in Table 1. We defined drugs as bacteriostatic or bactericidal based on annotation from Nichols et al [19]. All pairwise drug interaction experiments were done in duplicate, with correlation of 0.8 and 0.86 for *E*. *coli* and *A*. *baumannii* experiments, respectively (S10 Fig). We used the arithmetic average of two replicates as the drug interaction score for each pair.

LB media was prepared by dissolving 20g LB powder (Sigma) in 1l of water and autoclaving. Minimal media was prepared as final concentration of 1X M9 salts, 2uM MgSO4, 0.1uM CaCl2 and carbon source in water, and filtered for sterilization. A final concentration of 0.04% Glucose or 0.08% Glycerol was made, which makes the carbon resource levels equivalent in two media. 5ml bacteria cultures were grown in 15ml breathable culture tubes for 16 hours, by mixing 20ul of 25% glycerol stock of cells at OD600 = 1 and 5ml respective growth media, and shaking at 250RPM at 37C. After overnight growth, cells were diluted to OD = 0.01 and used as inoculums for the drug interaction experiments.

Drugs were dissolved in dimethyl sulfoxide and stored at −20°C. Nanoliter volumes of drugs and their combinations were printed on 384-well plates using a digital drug dispenser (D300e Digital Dispenser, HP). All drug sensitivity quantifications were done by measuring the optical density (OD600) of 50ul bacteria grown for 16 hours at 37C without shaking in 384-well plates (Synergy HT, BioTek). Dispense locations were randomized within each plate to minimize plate position effects. Plates were sealed with aluminum plate seals and incubated without shaking at 37°C. After data collection, plate data were reconstructed from randomized positions for further analysis.

### Prediction of drug interactions using MAGENTA

The entire series of steps to predict drug interactions using MAGENTA is described in S4 Fig. The inputs for MAGENTA were chemogenomic profiles of drugs and media conditions from Nichols et al [19], and log2 transformed drug interaction FIC scores for the training set. Chemogenomic data was quantile-normalized using the quantilenorm function in MATLAB. Interactions with chemogenomics fitness score less than -2 (two standard deviations below the mean) or greater than +2 were chosen to be significant and used as input to MAGENTA.

MAGENTA represents each drug *in silico* as a function of its corresponding drug-gene interactions inferred from chemogenomic profiling. MAGENTA assumes that cellular response to a combination of stressors can be represented as a linear combination of cellular response to individual stressors, as observed in prior studies [46,47]. This enables it to predict drug-drug interaction outcomes across metabolic conditions from individual chemogenomic profiles using the machine learning algorithm–Random forests. The random forest algorithm creates an ensemble of decision trees and outputs the mean prediction of the individual trees [48,49]. We used the RandomForest toolbox in MATLAB. The regression random forest algorithm was used with default parameters—500 trees (default) and number of variables sampled (default value–N/3, where N is the number of variables).

In addition to the test-set predictions, MAGENTA’s predictive ability was also assessed through tenfold cross-validation. In tenfold cross-validation, 10% of the interactions were randomly blinded and predicted by the model based on information from the remaining 90% of the interactions (S5 Fig). Through tenfold cross validation, we found that MAGENTA could accurately predict interactions with compounds that belong to novel chemical classes or with distinct mechanisms of action. Nevertheless, we found that the prediction accuracy could be further improved by choosing drugs and metabolic conditions representative of different classes in the training set.

For predicting interactions in *A*. *baumannii* using the orthology mapping approach, orthologous genes in *E*. *coli* were obtained from OrtholugeDB. 1633 genes were predicted to be orthologs of *A*. *baumannii* among the *E*. *coli* genes based on the reciprocal-best-BLAST-hit procedure.

The top genes predicted by MAGENTA to account for 75% of the model’s predictive ability were used for pathway enrichment analysis. KEGG annotations for E. coli were downloaded using the R Bioconductor GAGE Package. All statistical tests of correlation and overlap, and Multi-dimensional scaling analysis were performed in MATLAB. The MATLAB implementation of MAGENTA along with associated experimental data are provided as supplementary materials.

### Alternate metrics to evaluate predictive ability of MAGENTA

#### Area under the receiver-operating characteristics curve (AUC)

We also assessed the overall accuracy of MAGENTA by calculating the sensitivity (the true positive rate) and specificity (true negative rate) of the predictions, and by measuring the area under the receiver-operating characteristics curve (AUC). In a ROC curve, Sensitivity is plotted against the Specificity for different cut-offs for synergy or antagonism predicted by the model. To calculate these parameters, predictions and experimental observations were grouped into synergistic/antagonistic categories. The significance of the consistency was compared with a null model to assess statistical significance. The null model was obtained through 1,000 random permutations from the training data set and p-values were determined using a t-test.

We have performed this analysis with both an absolute cut off for experimentally observed synergy and antagonism (i.e. FIC < -0.2 for Synergy and FIC > 0.2 for Antagonism), and a relative cut offs (top and bottom 10%) for each dataset. MAGENTA performs equally well with both approaches. This suggests that MAGENTA predicts both relative order and the magnitude of change.

MAGENTA quantitatively predicted drug interactions with high sensitivity and specificity, significantly better than random, and with equal accuracy for both synergy and antagonism. For the three-way interaction predictions, MAGENTA achieved an AUC of 0.72 (p-value = 0.06) and 0.85 (p-value = 0.03) for top 10% (< -0.07) and hard threshold of -0.2 respectively for synergistic combinations. The corresponding values for antagonism were 0.76 (p-value = 0.01) and 0.8 (p-value = 0.002) for top 10% (> 0.9) and hard threshold of +0.2 respectively (S7 Fig).

Similarly, MAGENTA achieved a significantly high accuracy for predicting synergistic and antagonistic interactions in Glycerol media and for predicting interaction outcomes in *A*. *baumannii* (S7 Fig).

#### Comparison of mean and distribution of predicted and experimental interaction scores

Since correlation and ANOVA metric are insensitive to the range of the two variables that are being compared, we also assessed the similarity of the overall mean and distributions of the predicted and experimental interaction scores to benchmark the accuracy of MAGENTA. Comparison of the distribution of interaction scores in glycerol media suggests that MAGENTA can accurately predict the underlying distribution. There was no difference between the two distributions as assessed by t-test (p-value = 0.94) or non-parametric KS test (p-value = 0.3). The overall mean of MAGENTA predictions was similar to the experimental measurements (S8 Fig). Similarly, interaction predictions for Acinetobacter had a similar distribution to that of the experimental data. There was no difference between the two distributions as assessed by t-test (p-value = 0.44) or non-parametric KS test (p-value = 0.94) (S8 Fig).

For the three-way drug interaction predictions, MAGENTA was trained on interaction data from our prior study which used alpha scores instead of FIC to quantify the interactions. Alpha scores have a larger range and variance (variance = 2.02 and 0.12, mean = 1.13 and 0.28, for alpha scores and FIC scores in LB media). To control for the differences in distribution, we re-scaled the output predictions to have the same distribution as FIC scores from LB media. This transformation doesn’t change the order of our predicted scores, hence we got the same correlation, but the mean and distribution were more similar to the experimental data (S8 Fig). Since predictions in glycerol media and in *A*. *baumannii* were trained on both alpha and FIC scores generated as training data in this study, the distribution was similar to the experimental set, which was measured using the FIC metric. Therefore, MAGENTA captures both range and order if it is trained and tested using the same metrics. In cases where different metrics are used, the relative order of MAGENTA predictions are conserved i.e. interactions predicted to be most synergistic will be most synergistic across other metrics used for experimental quantification.

#### Comparison with three-way interaction predictions using pairwise interaction data

To benchmark the accuracy of model predictions for three-way interactions without using chemogenomics data, we used pairwise combinations of underlying drugs to predict triplet combinations. Averaging pairwise interactions has been used previously to make successful predictions for 3-way interactions [35,36]. The advantage of MAGENTA is that it can make predictions for combinations with new drugs that it was not trained on using chemogenomics data. In our case, three of 8 the tested drugs are not part of the training set. Hence, out of the 56 triplet combinations, only 10 triplets can be predicted using pairwise data in the training set. The 10 predictions by averaging correlated with the 3-way data with R = 0.74 (p = 0.01). MAGENTA predictions for 3-way combinations also correlated significantly with experimental values for the 56 combinations with R = 0.57 (p < 10^−6^). Using our training data from our prior study involving interactions between 19 drugs and chemogenomics data for 72 drugs, MAGENTA can provide predictions for 59,640 3-way combinations, while averaging provides predictions for only 969 3-way combinations. While, the averaging method has a slightly higher correlation than MAGENTA, the number of interactions predicted by MAGENTA is an order of magnitude higher than the averaging method. Therefore, in contrast to the averaging method, MAGENTA can predict a significantly larger number of interactions, can predict drug interaction changes in different metabolic conditions, and provides biological insight on the observed interaction.

Finally, we used a null model to benchmark the accuracy of MAGENTA triplet predictions. We split the model predictions and experimental data as synergistic (log2(FIC) < -0.2), neutral or antagonistic (> 0.2). We generated a null model based on random sampling from a trinomial distribution derived from the training set. We compared MAGENTA predictions with 10,000 random predictions with the same trinomial distribution as the training set. MAGENTA had a significantly higher accuracy than expected from a null model (p-value = 1 x 10^−7^).

## Supporting information

S1 Fig The Integration of multiple chemogenomic profiles in MAGENTA. To re-scale the scores for a multi-drug combination, we normalize them by the number of drugs in a combination in order to achieve the same units as the model defined for two drugs. The multiplier (2/3) used in the above figure is the scaling factor used for a three-drug combination. In general, the scores are multiplied by 2/N where N is the number of drugs in a combination. MAGENTA then compares the joint chemogenomic profile with the drug interaction score to identify genes predictive of drug interaction outcome.
(TIF)

S2 Fig Multi-dimensional scaling analysis shows that the chemogenomic profiles of metabolic conditions differ from the profiles of drugs. The profiles of metabolic conditions are shown in red. The outlier ethanol is clustered with drugs because it also causes cellular stress in addition to being a nutrient. Pearson’s correlation was used as the distance metric for visualization.
(TIF)

S3 Fig MAGENTA accurately predicts multi-drug interaction outcomes. We assessed MAGENTA predictions using the Anova statistic. Experimental interaction scores were classified as strongly synergistic (log-FIC < -0.2, N = 2), neutral or antagonistic (log-FIC > 0.2, N = 45). The box plot shows the predicted interaction scores by MAGENTA for each of these three classes. Comparison of predicted scores with the experimental scores revealed that the predicted scores differed significantly between the three classes (Anova p-value = 0.0003).
(TIF)

S4 Fig Overview of the steps to predict drug interactions using MAGENTA. (TIF)

S5 Fig Testing MAGENTA’s predictive ability through tenfold cross-validation. In tenfold cross-validation, 10% of all interactions used for training MAGENTA in LB, Glucose and Glycerol media were randomly removed, and their interaction scores were predicted by the MAGENTA based on information from the remaining 90% of the interactions. The plot shows that MAGENTA accurately predicted interaction outcomes in all ten rounds of cross validation (mean rank correlation R = 0.71)
(TIF)

S6 Fig Distribution of interaction outcomes across the three media conditions shows the shift towards synergy in glucose and glycerol conditions. (TIF)

S7 Fig Sensitivity vs specificity curves for MAGENTA predictions of synergy and antagonism. Sensitivity measures the true positive rate, which is the fraction of true positive interactions correctly identified; specificity measures the true negative rate. the area under the ROC curve (AUC) values were determined by the *prefcurve* function in MATLAB, which measures sensitivity and specificity of model predictions over a range of thresholds. A and B. Predictions of synergy and antagonism for Triplet combinations using relative threshold (Panel A) and hard threshold (Panel B). AUC synergy = 0.72 (p-value = 0.06) and 0.85 (p-value = 0.03) for top 10% (< -0.07) and hard threshold of -0.2 respectively. AUC antagonism = 0.76 (p-value = 0.01) and 0.8 (p-value = 0.002) for top 10% (> 0.9) and hard threshold of +0.2 respectively. C and D. Predictions of synergy and antagonism in Glycerol media using relative threshold (Panel C) and hard threshold (Panel D). AUC synergy = 0.77 (p-value = 0.02) and 0.78 (p-value = 3.5 x 10^−4^) for top 10% (< -0.88) and hard threshold of -0.2 respectively. AUC antagonism = 0.79 (p-value = 0.019) and 0.86 (p-value = 5 x 10^−6^) for top 10% (> 0.78) and hard threshold of +0.2 respectively. E and F. The ROC curves for predictions in *A*. *baumannii* using relative (panel E) and absolute threshold (panel F) for synergy and antagonism. AUC synergy = 0.94 (p-value = 0.002) and 0.81 (p-value = 0.0003) for top 10% (< -0.95) and hard threshold of -0.2 respectively. AUC antagonism = 0.74 (p-value = 0.01) and 0.69 (p-value = 0.16) for top 10% (> 0.44) and hard threshold of +0.2 respectively. P-values were estimated by comparison with 1,000 random permutations from the training data set using a t-test.
(TIF)

S8 Fig Distribution of predicted and experimental interaction outcomes shows that MAGENTA can accurately predict the overall distribution. Comparison with non-parametric KS test shows that there was no significant difference between the two predicted and observed distributions (p-value > 0.05). A. Distribution of interaction outcomes for MAGENTA and experimental observation for three-way combinations after normalization of output predictions. B. Distribution of interaction outcomes for MAGENTA and experimental observation in glycerol media. C. Distribution of interaction outcomes for MAGENTA and experimental observation in *A*. *baumannii*.
(TIF)

S9 Fig The drug-drug interaction landscape of *M. tuberculosis* and *S. aureus*. The heat maps show the predicted impact of 9 distinct metabolic environments on the interaction outcomes of 2556 pairwise drug combinations (synergy (red), antagonism (blue)). The drug combinations and metabolic conditions are clustered based on similarity. Panel a and b shows the corresponding interaction scores for *M*. *tuberculosis and S*. *aureus* respectively.
(TIF)

S10 Fig Correlation between replicates for the experimental drug interaction screens. The rank correlation and the corresponding p-value are shown in each plot. We used the arithmetic average of two replicates as the drug interaction score for each pair.
(TIF)

S11 Fig Correlation between replicates for the E. coli drug interaction dataset given in Fig 1. Choosing IC 50–80 (i.e. 50–80% inhibition) gives highly reproducible interaction scores; hence we used IC70 for calculating interaction scores.
(TIF)

S12 Fig The plot shows the agreement between interaction scores obtained using different IC levels in the *E. coli* drug interaction data set. Choosing IC 50–80 gives robust interaction scores with correlation R > 0.8.
(TIF)

S1 Table Top 10 genes used by MAGENTA model to make predictions in new media conditions. (PDF)

S2 Table Top 10 genes used by MAGENTA model to make triplets predictions. (PDF)

S3 Table Top 10 pathways used by MAGENTA model to make triplets predictions. (PDF)

S4 Table Top 10 pathways enriched in the chemogenomic profile of glucose media relative to the rest of the genes in the chemogenomics data. (PDF)

S5 Table Conservation of top predictive genes in MAGENTA (319 genes that explain 75% of predictive ability) between *E. coli* and *A. baumannii*. The table shows enriched pathways among the genes that were not conserved in *A*. *baumanni* (table A) and those that were conserved (table B). Top 10 pathways sorted based on p-value are shown (hypergeometric p-value < 0.05).
(PDF)

S6 Table Drug combinations that showed robust synergy across all metabolic conditions in *E. coli* and *A. baumannii*. Analysis of 2556 drug combinations revealed 19 combinations in *A*. *baumannii* and 119 combinations in *E*. *coli* listed above that showed synergy across all the growth conditions (Interaction score < -0.5).
(PDF)

S7 Table Drug combinations that showed robust synergy across all metabolic conditions in *M. tuberculosis and S. aureus*. Analysis of 2556 drug combinations revealed 113 combinations in *M*. *tuberculosis* and 108 combinations in *S*. *aureus* listed above that showed synergy across all the growth conditions (Interaction score < -0.5).
(PDF)

S1 Text Contents in supplementary dataset. (PDF)
