# Supplementary figures and images for: Chemogenomic model identifies synergistic drug combinations robust to the pathogen microenvironment
Source: PLoS Comput Biol. 2018 Dec 31;14(12):e1006677. doi: 10.1371/journal.pcbi.1006677 (PMC6329523; doi:10.1371/journal.pcbi.1006677)

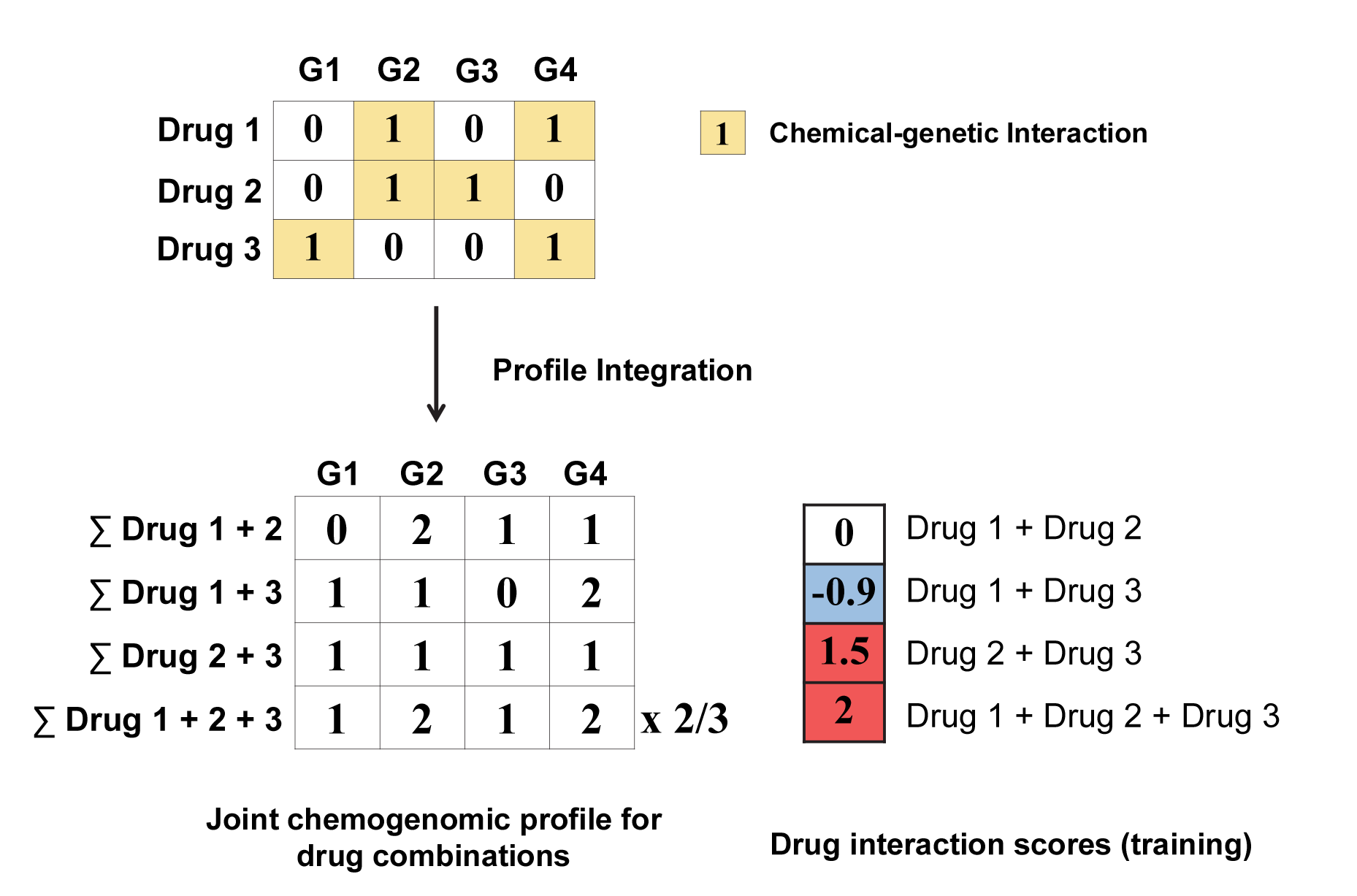

Supplement: S1 Fig — To re-scale the scores for a multi-drug combination, we normalize them by the number of drugs in a combination in order to achieve the same units as the model defined for two drugs. The multiplier (2/3) used in the above figure is the scaling factor used for a three-drug combination. In general, the scores are multiplied by 2/N where N is the number of drugs in a combination. MAGENTA then compares the joint chemogenomic profile with the drug interaction score to identify genes predictive of drug interaction outcome. (TIF) [file pcbi.1006677.s001.tif]

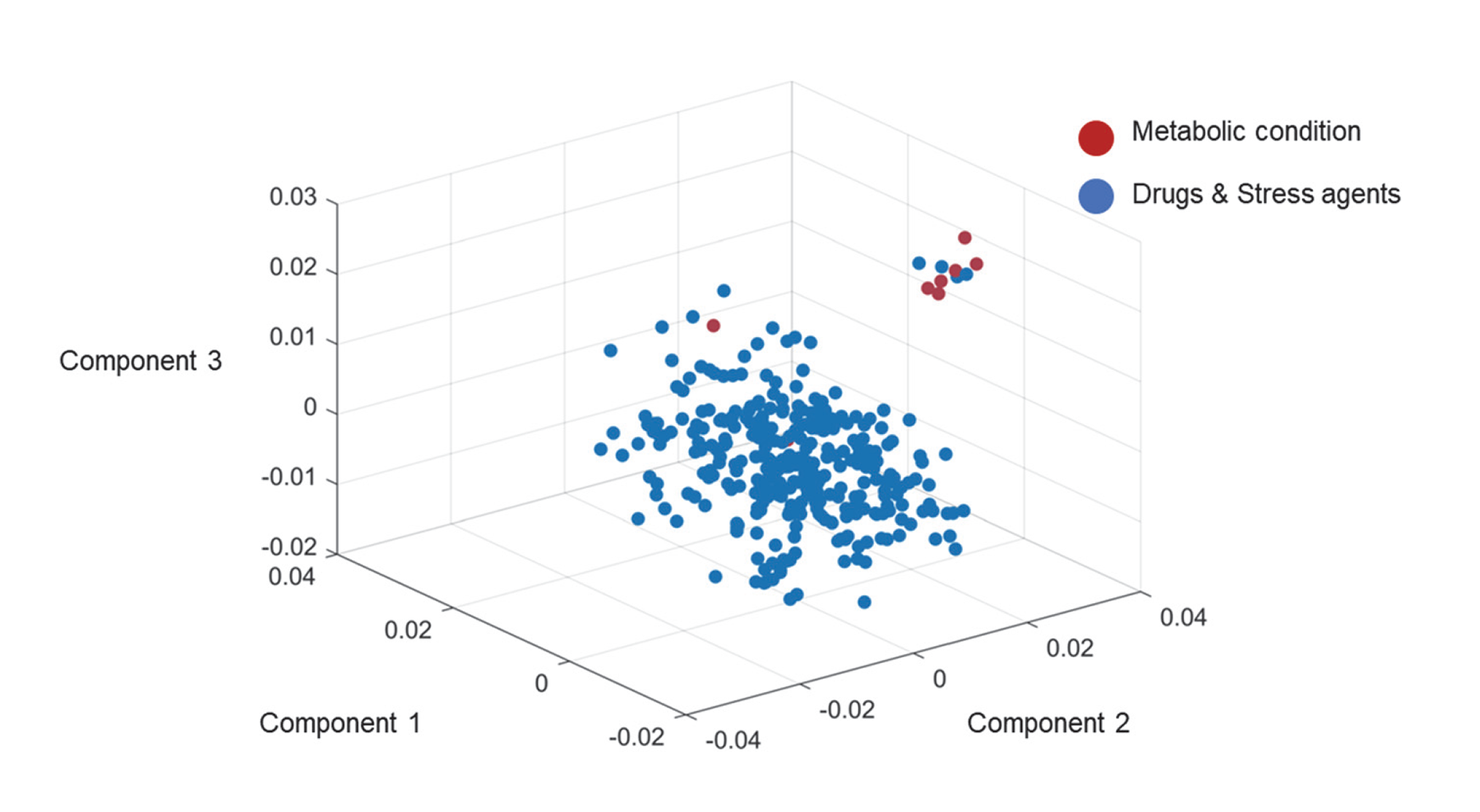

Supplement: S2 Fig — The profiles of metabolic conditions are shown in red. The outlier ethanol is clustered with drugs because it also causes cellular stress in addition to being a nutrient. Pearson’s correlation was used as the distance metric for visualization. (TIF) [file pcbi.1006677.s002.tif]

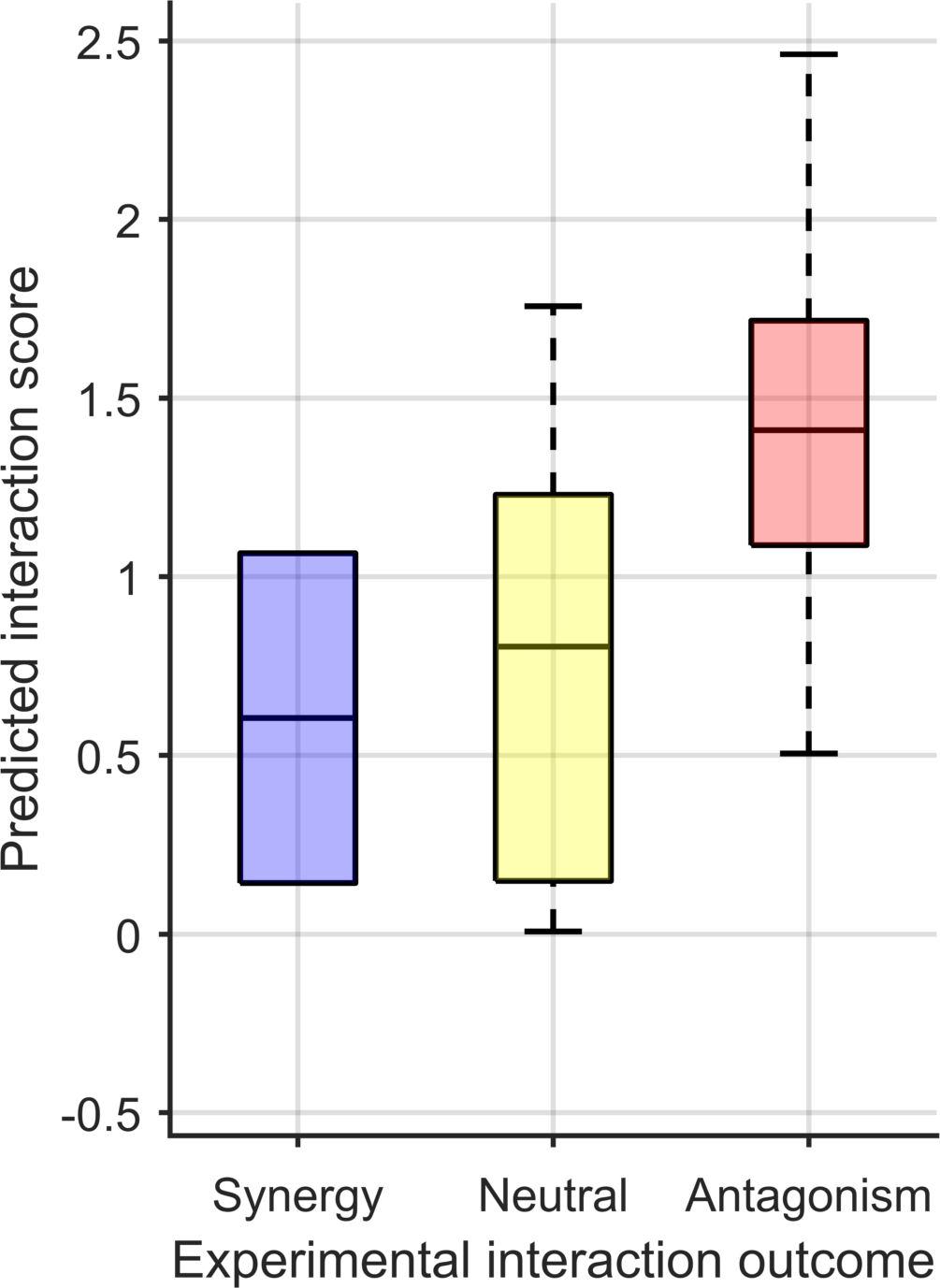

Supplement: S3 Fig — We assessed MAGENTA predictions using the Anova statistic. Experimental interaction scores were classified as strongly synergistic (log-FIC < -0.2, N = 2), neutral or antagonistic (log-FIC > 0.2, N = 45). The box plot shows the predicted interaction scores by MAGENTA for each of these three classes. Comparison of predicted scores with the experimental scores revealed that the predicted scores differed significantly between the three classes (Anova p-value = 0.0003). (TIF) [file pcbi.1006677.s003.tif]

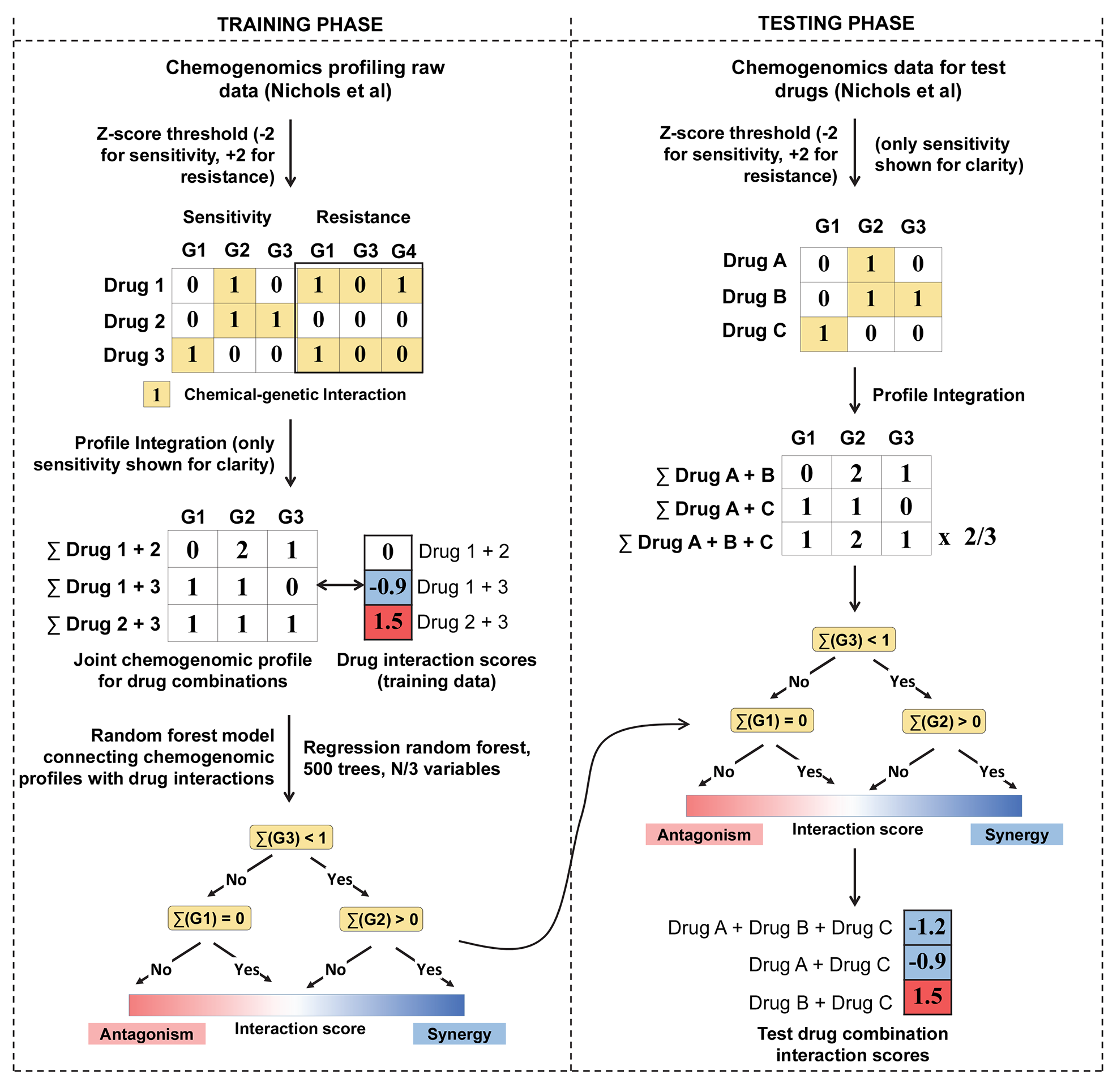

Supplement: S4 Fig — (TIF) [file pcbi.1006677.s004.tif]

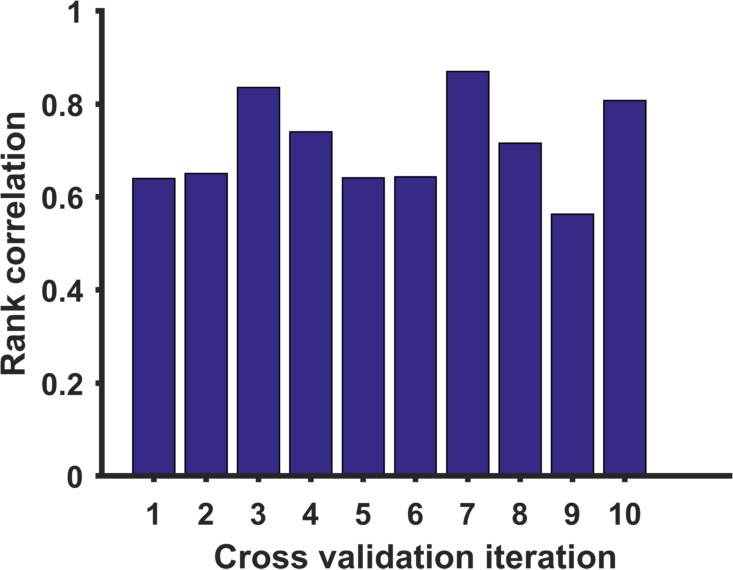

Supplement: S5 Fig — In tenfold cross-validation, 10% of all interactions used for training MAGENTA in LB, Glucose and Glycerol media were randomly removed, and their interaction scores were predicted by the MAGENTA based on information from the remaining 90% of the interactions. The plot shows that MAGENTA accurately predicted interaction outcomes in all ten rounds of cross validation (mean rank correlation R = 0.71) (TIF) [file pcbi.1006677.s005.tif]

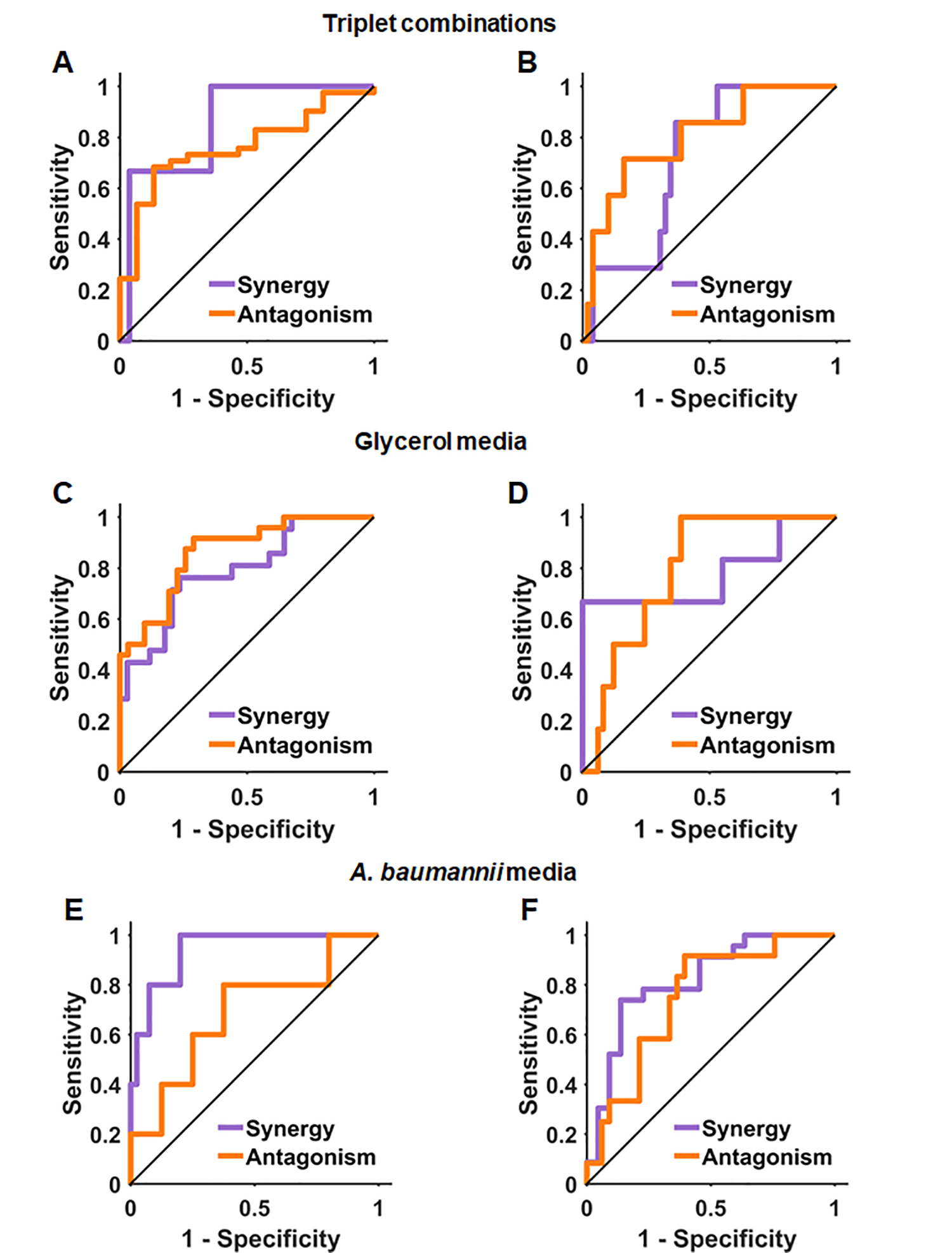

Supplement: S7 Fig — Sensitivity measures the true positive rate, which is the fraction of true positive interactions correctly identified; specificity measures the true negative rate. the area under the ROC curve (AUC) values were determined by the prefcurve function in MATLAB, which measures sensitivity and specificity of model predictions over a range of thresholds. A and B. Predictions of synergy and antagonism for Triplet combinations using relative threshold (Panel A) and hard threshold (Panel B). AUC synergy = 0.72 (p-value = 0.06) and 0.85 (p-value = 0.03) for top 10% (< -0.07) and hard threshold of -0.2 respectively. AUC antagonism = 0.76 (p-value = 0.01) and 0.8 (p-value = 0.002) for top 10% (> 0.9) and hard threshold of +0.2 respectively. C and D. Predictions of synergy and antagonism in Glycerol media using relative threshold (Panel C) and hard threshold (Panel D). AUC synergy = 0.77 (p-value = 0.02) and 0.78 (p-value = 3.5 x 10^−4^) for top 10% (< -0.88) and hard threshold of -0.2 respectively. AUC antagonism = 0.79 (p-value = 0.019) and 0.86 (p-value = 5 x 10^−6^) for top 10% (> 0.78) and hard threshold of +0.2 respectively. E and F. The ROC curves for predictions in A. baumannii using relative (panel E) and absolute threshold (panel F) for synergy and antagonism. AUC synergy = 0.94 (p-value = 0.002) and 0.81 (p-value = 0.0003) for top 10% (< -0.95) and hard threshold of -0.2 respectively. AUC antagonism = 0.74 (p-value = 0.01) and 0.69 (p-value = 0.16) for top 10% (> 0.44) and hard threshold of +0.2 respectively. P-values were estimated by comparison with 1,000 random permutations from the training data set using a t-test. (TIF) [file pcbi.1006677.s007.tif]

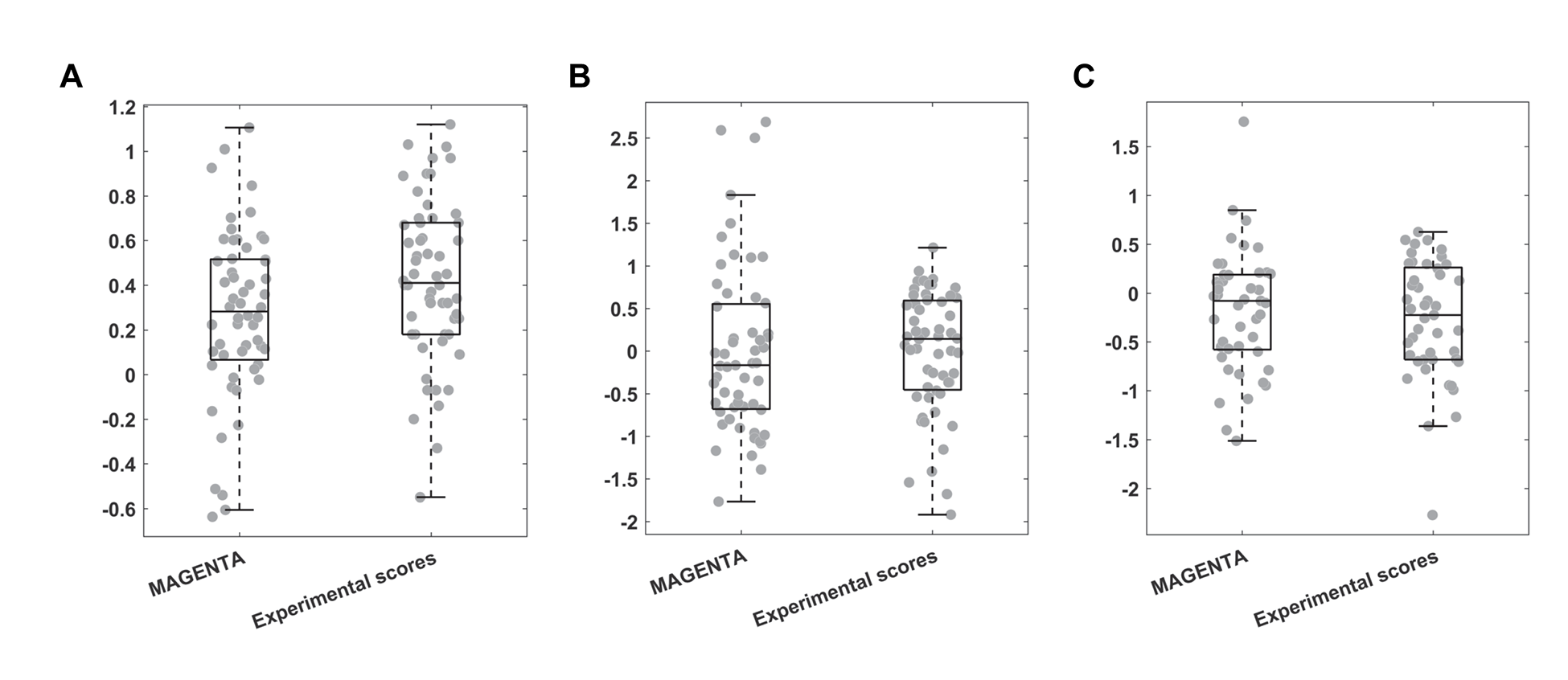

Supplement: S8 Fig — Comparison with non-parametric KS test shows that there was no significant difference between the two predicted and observed distributions (p-value > 0.05). A. Distribution of interaction outcomes for MAGENTA and experimental observation for three-way combinations after normalization of output predictions. B. Distribution of interaction outcomes for MAGENTA and experimental observation in glycerol media. C. Distribution of interaction outcomes for MAGENTA and experimental observation in A. baumannii. (TIF) [file pcbi.1006677.s008.tif]

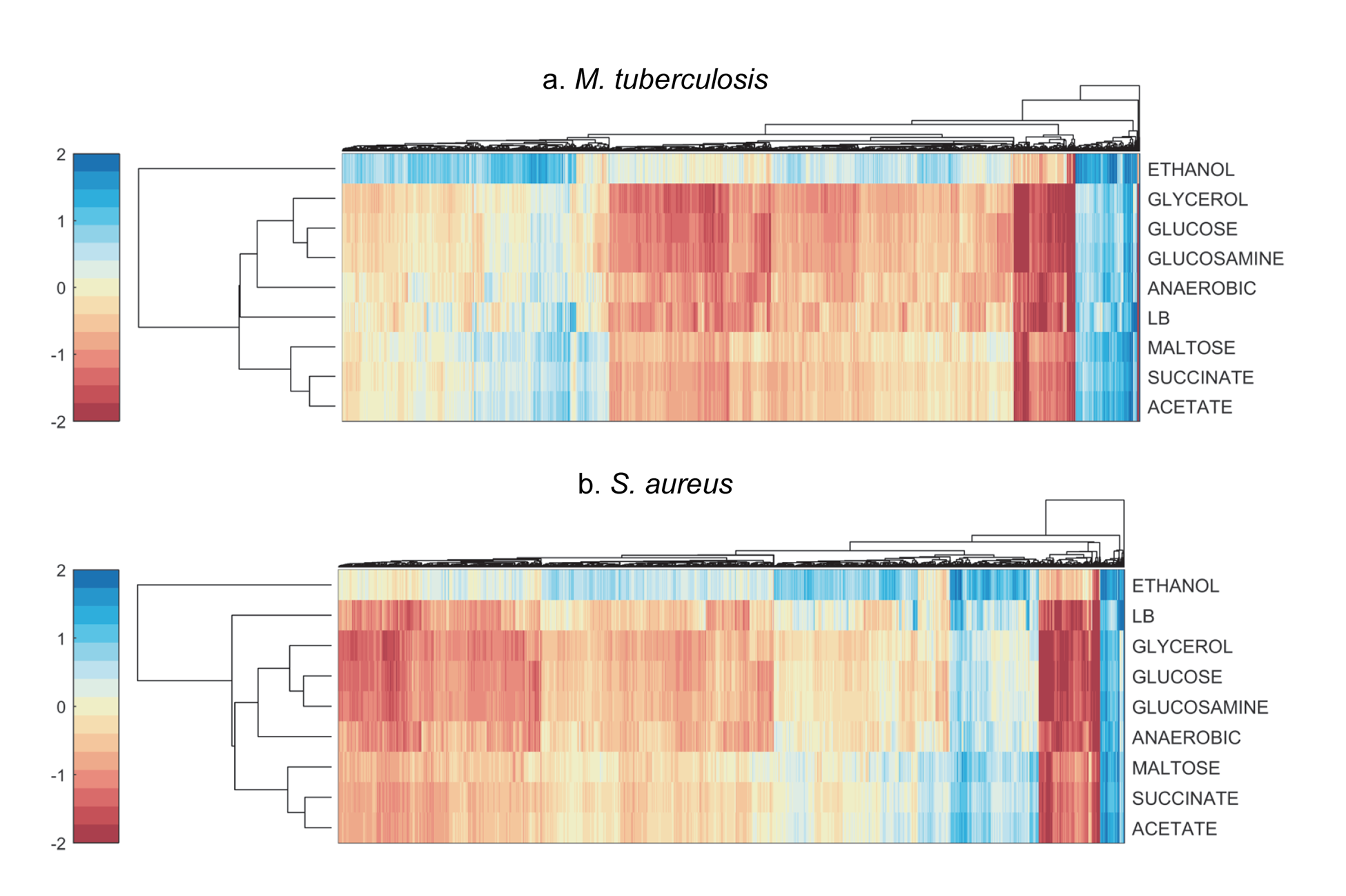

Supplement: S9 Fig — The heat maps show the predicted impact of 9 distinct metabolic environments on the interaction outcomes of 2556 pairwise drug combinations (synergy (red), antagonism (blue)). The drug combinations and metabolic conditions are clustered based on similarity. Panel a and b shows the corresponding interaction scores for M. tuberculosis and S. aureus respectively. (TIF) [file pcbi.1006677.s009.tif]

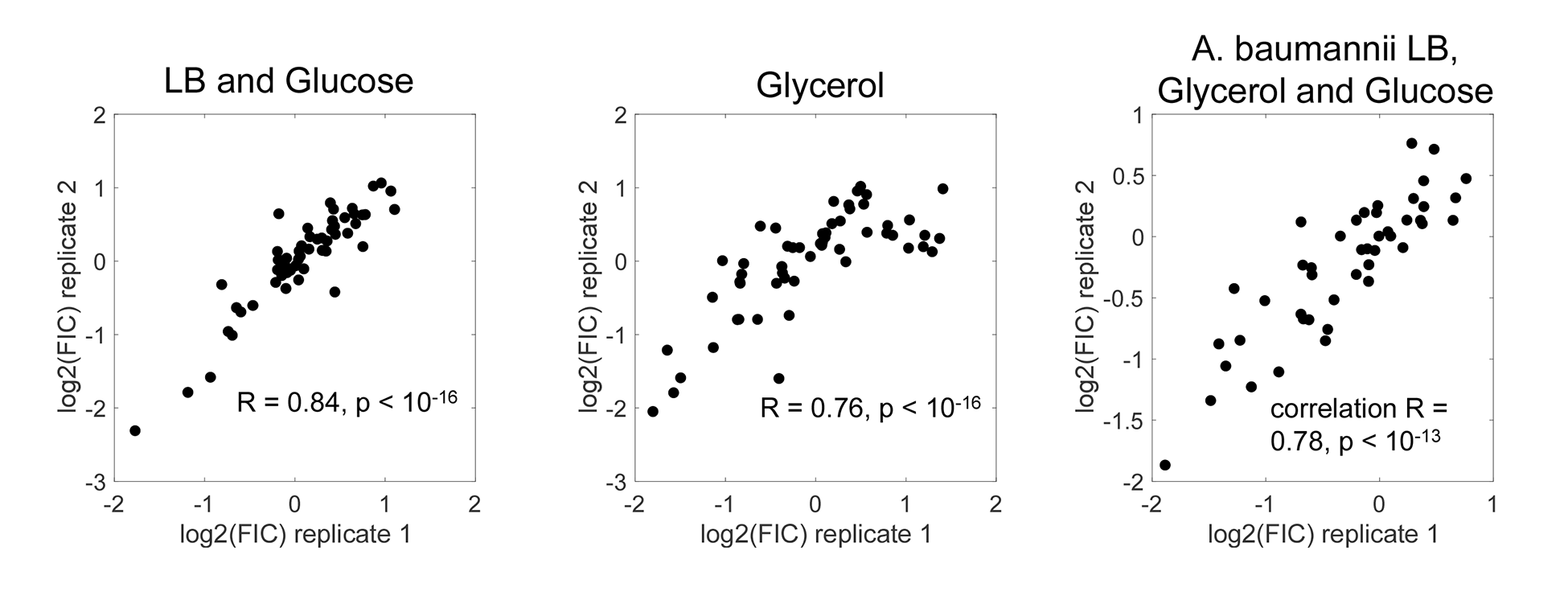

Supplement: S10 Fig — The rank correlation and the corresponding p-value are shown in each plot. We used the arithmetic average of two replicates as the drug interaction score for each pair. (TIF) [file pcbi.1006677.s010.tif]

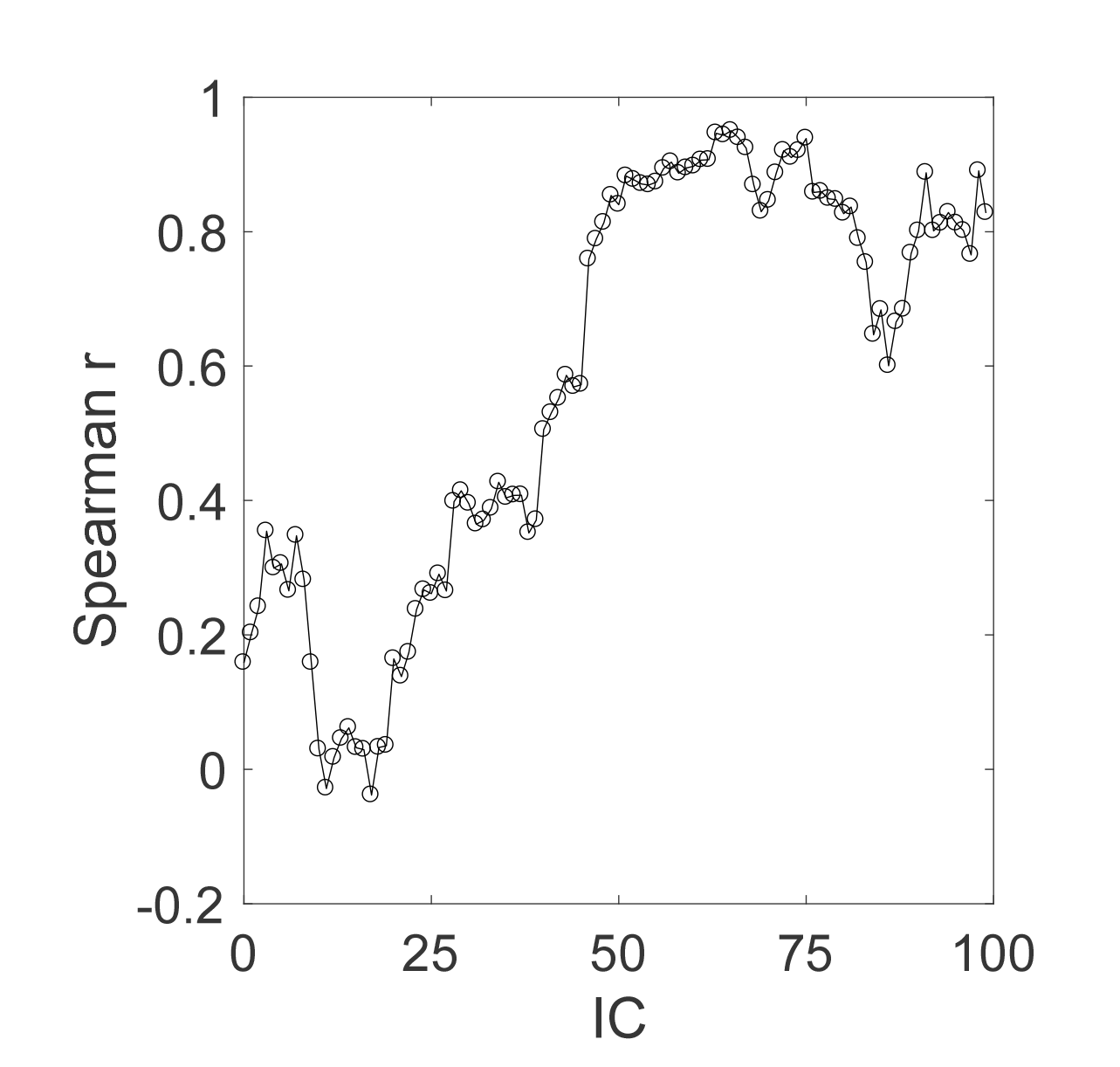

Supplement: S11 Fig — Choosing IC 50–80 (i.e. 50–80% inhibition) gives highly reproducible interaction scores; hence we used IC70 for calculating interaction scores. (TIF) [file pcbi.1006677.s011.tif]

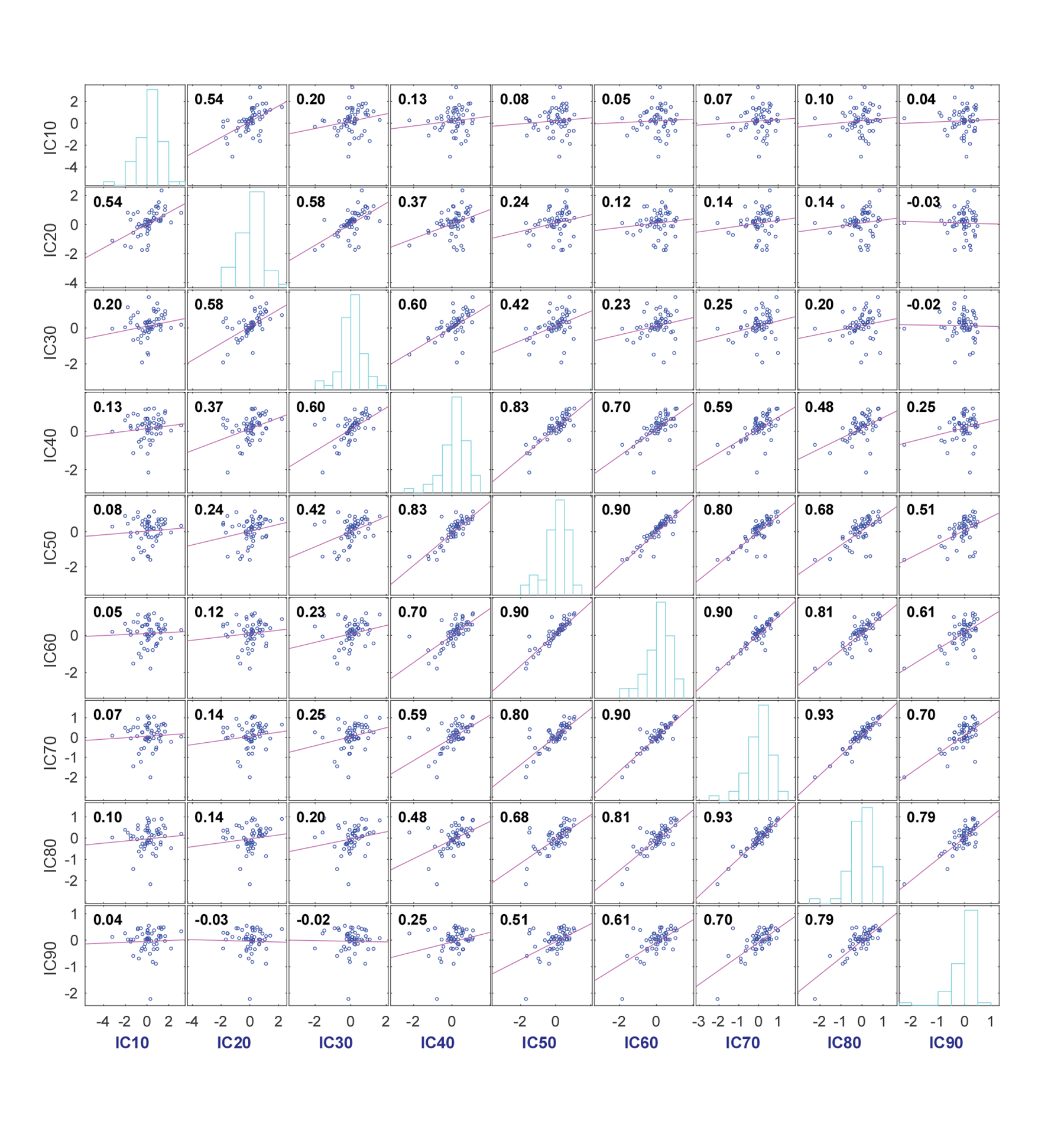

Supplement: S12 Fig — Choosing IC 50–80 gives robust interaction scores with correlation R > 0.8. (TIF) [file pcbi.1006677.s012.tif]
