# Supplementary material for: Chemogenomic model identifies synergistic drug combinations robust to the pathogen microenvironment
Source: PLoS Comput Biol. 2018 Dec 31;14(12):e1006677. doi: 10.1371/journal.pcbi.1006677 (PMC6329523; doi:10.1371/journal.pcbi.1006677)
Supplement: S1 Table — (PDF) [file pcbi.1006677.s013.pdf]

yjjY	Uncharacterized protein YjjY
elfD	Probable fimbrial chaperone protein ElfD
yfbN	Uncharacterized protein YfbN
glnA	Glutamine synthetase (GS) (EC 6.3.1.2) (Glutamate--ammonia ligase)
yfjH	Uncharacterized protein YfjH
ruvC	Crossover junction endodeoxyribonuclease RuvC (EC 3.1.22.4)
glmS	Glutamine--fructose-6-phosphate aminotransferase
ruvA	Holliday junction ATP-dependent DNA helicase RuvA (EC 3.6.4.12)
recC	RecBCD enzyme subunit RecC (EC 3.1.11.5)
ybeD	UPF0250 protein YbeD
