# Supplementary material for: Chemogenomic model identifies synergistic drug combinations robust to the pathogen microenvironment
Source: PLoS Comput Biol. 2018 Dec 31;14(12):e1006677. doi: 10.1371/journal.pcbi.1006677 (PMC6329523; doi:10.1371/journal.pcbi.1006677)
Supplement: S2 Table — (PDF) [file pcbi.1006677.s014.pdf]

glmS	Glutamine--fructose-6-phosphate aminotransferase
ubiX	Flavin prenyltransferase UbiX (EC 2.5.1.129)
tdk	Thymidine kinase (EC 2.7.1.21)
elfD	Probable fimbrial chaperone protein ElfD
yjjY	Uncharacterized protein YjjY
yfbN	Uncharacterized protein YfbN
ycgG	Probable cyclic di-GMP phosphodiesterase PdeG (EC 3.1.4.52)
rpmE	50S ribosomal protein L31 (Large ribosomal subunit protein bL31-A)
ybfC	Uncharacterized protein YbfC
xseA	Exodeoxyribonuclease 7 large subunit (EC 3.1.11.6)
