# Supplementary material for: Chemogenomic model identifies synergistic drug combinations robust to the pathogen microenvironment
Source: PLoS Comput Biol. 2018 Dec 31;14(12):e1006677. doi: 10.1371/journal.pcbi.1006677 (PMC6329523; doi:10.1371/journal.pcbi.1006677)
Supplement: S3 Table — (PDF) [file pcbi.1006677.s015.pdf]

Pathways	Total genes	P-value
Alanine, aspartate and glutamate metabolism	8	0.0003
Homologous recombination	7	0.001
Oxidative phosphorylation	9	0.001
beta-Lactam resistance	5	0.001
Mismatch repair	5	0.005
Glycerolipid metabolism	3	0.013
Lipopolysaccharide biosynthesis	5	0.013
Galactose metabolism	6	0.014
Pyrimidine metabolism	9	0.018
Glyoxylate and dicarboxylate metabolism	6	0.021
Streptomycin biosynthesis	2	0.025
C5-Branched dibasic acid metabolism	2	0.034
