# Supplementary material for: Chemogenomic model identifies synergistic drug combinations robust to the pathogen microenvironment
Source: PLoS Comput Biol. 2018 Dec 31;14(12):e1006677. doi: 10.1371/journal.pcbi.1006677 (PMC6329523; doi:10.1371/journal.pcbi.1006677)
Supplement: S4 Table — (PDF) [file pcbi.1006677.s016.pdf]

Pathways	Total genes	P-value
Lysine biosynthesis	3	6.93E-05
Aminoacyl-tRNA biosynthesis	2	0.000134
Folate biosynthesis	3	0.000302
Sulfur relay system	3	0.00113
Two-component system	10	0.004256
Amino sugar and nucleotide sugar metabolism	4	0.00451
Pentose phosphate pathway	3	0.009364
beta-Lactam resistance	2	0.011684
Phenylalanine, tyrosine and tryptophan biosynthesis	2	0.025993
ABC transporters	9	0.02702
Fructose and mannose metabolism	3	0.031405
