# Supplementary material for: Chemogenomic model identifies synergistic drug combinations robust to the pathogen microenvironment
Source: PLoS Comput Biol. 2018 Dec 31;14(12):e1006677. doi: 10.1371/journal.pcbi.1006677 (PMC6329523; doi:10.1371/journal.pcbi.1006677)
Supplement: S5 Table — The table shows enriched pathways among the genes that were not conserved in A. baumanni (table A) and those that were conserved (table B). Top 10 pathways sorted based on p-value are shown (hypergeometric p-value < 0.05). (PDF) [file pcbi.1006677.s017.pdf]

A. Enriched KEGG pathways among non-orthologous genes	p-value
eco00540 Lipopolysaccharide biosynthesis	0.000324
eco00521 Streptomycin biosynthesis	0.003221
eco03430 Mismatch repair	0.0212
eco00040 Pentose and glucuronate interconversions	0.025326
eco00500 Starch and sucrose metabolism	0.043669

B. Enriched KEGG pathways among orthologous genes	p-value
eco00190 Oxidative phosphorylation	4.40E-10
eco00250 Alanine, aspartate and glutamate metabolism	3.71E-07
eco00230 Purine metabolism	1.38E-05
eco03440 Homologous recombination	3.24E-05
eco00400 Phenylalanine, tyrosine and tryptophan biosynthesis	9.17E-05
eco00260 Glycine, serine and threonine metabolism	0.000108
eco00340 Histidine metabolism	0.000154
eco00473 D-Alanine metabolism	0.000246
eco00750 Vitamin B6 metabolism	0.000268
eco00240 Pyrimidine metabolism	0.000889
