# Supplementary material for: Chemogenomic model identifies synergistic drug combinations robust to the pathogen microenvironment
Source: PLoS Comput Biol. 2018 Dec 31;14(12):e1006677. doi: 10.1371/journal.pcbi.1006677 (PMC6329523; doi:10.1371/journal.pcbi.1006677)
Supplement: S6 Table — Analysis of 2556 drug combinations revealed 19 combinations in A. baumannii and 119 combinations in E. coli listed above that showed synergy across all the growth conditions (Interaction score < -0.5). (PDF) [file pcbi.1006677.s018.pdf]

A. baumannii	*E. coli*
ActinomycinD + Fusidic acid	A22 + Fusidicacid
Amoxicillin + Fusidic acid	ActinomycinD + Fusidicacid
Ampicillin + Aztreonam	Amikacin + Ampicillin
Ampicillin + Fusidic acid	Amikacin + Cisplatin
Azithromycin + Fusidic acid	Amikacin + Fusidicacid
Aztreonam + Fusidic acid	Ampicillin + Azithromycin
Carbenicillin + Fusidic acid	Ampicillin + Bacitracin
Ceftazidime + Fusidic acid	Ampicillin + Calcofluor
Clarythromycin + Fusidic acid	Ampicillin + Carbenicillin
EGCG+ Fusidic acid	Ampicillin + Cccp
Erythromycin + Fusidic acid	Ampicillin + Cisplatin
Fusidic acid+ Methotrexate	Ampicillin + Clarythromycin
Fusidic acid+ Nigericin	Ampicillin + EGCG
Fusidic acid+ Oxacillin	Ampicillin + Erythromycin
Fusidic acid+ Rifampicin	Ampicillin + Fusidicacid
Fusidic acid+ Spiramycin	Ampicillin + Indolicidin
Fusidic acid+ Tetracycline	Ampicillin + Methotrexate
Fusidic acid+ Vancomycin	Ampicillin + Nigericin
Tetracycline + Vancomycin	Ampicillin + Novobiocin
	Ampicillin + Oxacillin
	Ampicillin + Phleomycin
	Ampicillin + Radicicol
	Ampicillin + Rifampicin
	Ampicillin + Spiramycin
	Ampicillin + Sulfamethizole
	Ampicillin + Tetracycline
	Ampicillin + Tunicamycin
	Ampicillin + Vancomycin
	Ampicillin + Verapamil
	Azithromycin + Bacitracin
	Azithromycin + Carbenicillin
	Azithromycin + Cisplatin
	Azithromycin + Clarythromycin
	Azithromycin + Erythromycin
	Azithromycin + Fusidicacid
	Azithromycin + Novobiocin
	Azithromycin + Phleomycin
	Azithromycin + Rifampicin
	Azithromycin + Spiramycin
	Azithromycin + Tetracycline
	Azithromycin + Vancomycin
	Azithromycin + Verapamil
	Bacitracin+ Cisplatin
	Bacitracin+ Fusidicacid

	Bacitracin+ Tetracycline
	Bleomycin + Fusidicacid
	Calcofluor+ Cisplatin
	Calcofluor+ Fusidicacid
	Carbenicillin + Cisplatin
	Carbenicillin + Fusidicacid
	Carbenicillin + Tetracycline
	Cccp + Fusidicacid
	Cccp + Tetracycline
	Cefaclor + Fusidicacid
	Cefoxitin + Fusidicacid
	Cefsulodin + Fusidicacid
	Ceftazidime + Fusidicacid
	Chir090 + Fusidicacid
	Chloropromazine+ Fusidicacid
	Cisplatin + Clarythromycin
	Cisplatin + Fusidicacid
	Cisplatin + Methotrexate
	Cisplatin + Nigericin
	Cisplatin + Oxacillin
	Cisplatin + Rifampicin
	Cisplatin + Spiramycin
	Cisplatin + Sulfamethizole
	Cisplatin + Sulfamonomethoxine
	Cisplatin + Tetracycline
	Cisplatin + Vancomycin
	Clarythromycin + Fusidicacid
	Clarythromycin + Novobiocin
	Clarythromycin + Phleomycin
	Clarythromycin + Rifampicin
	Clarythromycin + Tetracycline
	Clarythromycin + Vancomycin
	Cycloserined+ Fusidicacid
	Dibucaine + Fusidicacid
	Doxorubicin + Fusidicacid
	Doxycycline + Fusidicacid
	EGCG+ Fusidicacid
	Erythromycin + Fusidicacid
	Erythromycin + Tetracycline
	Erythromycin + Vancomycin
	Fusidicacid + Hydroxyurea
	Fusidicacid + Indolicidin
	Fusidicacid + Isoniazid
	Fusidicacid + Mecillinam
	Fusidicacid + Methotrexate

	Fusidicacid + Minocycline
	Fusidicacid + Nigericin
	Fusidicacid + Norfloxacin
	Fusidicacid + Novobiocin
	Fusidicacid + Oxacillin
	Fusidicacid + Phleomycin
	Fusidicacid + Procaine
	Fusidicacid + Propidiumiodide
	Fusidicacid + Puromycin
	Fusidicacid + Radicicol
	Fusidicacid + Rifampicin
	Fusidicacid + Spectinomycin
	Fusidicacid + Spiramycin
	Fusidicacid + Streptonigrin
	Fusidicacid + Sulfamethizole
	Fusidicacid + Sulfamonomethoxine
	Fusidicacid + Tetracycline
	Fusidicacid + Triclosan
	Fusidicacid + Vancomycin
	Fusidicacid + Verapamil
	Novobiocin+ Spiramycin
	Novobiocin+ Tetracycline
	Novobiocin+ Vancomycin
	Oxacillin + Tetracycline
	Oxacillin + Vancomycin
	Phleomycin + Tetracycline
	Rifampicin + Tetracycline
	Spiramycin+ Tetracycline
	Tetracycline + Vancomycin
	Tetracycline + Verapamil
