# Supplementary material for: Chemogenomic model identifies synergistic drug combinations robust to the pathogen microenvironment
Source: PLoS Comput Biol. 2018 Dec 31;14(12):e1006677. doi: 10.1371/journal.pcbi.1006677 (PMC6329523; doi:10.1371/journal.pcbi.1006677)
Supplement: S7 Table — Analysis of 2556 drug combinations revealed 113 combinations in M. tuberculosis and 108 combinations in S. aureus listed above that showed synergy across all the growth conditions (Interaction score < -0.5). (PDF) [file pcbi.1006677.s019.pdf]

S. aureus	*M. tuberculosis*
A22 + Fusidicacid	A22 + Fusidicacid
Actinomycind + Ampicillin	Actinomycind + Ampicillin
Actinomycind + Cisplatin	Actinomycind + Cisplatin
Actinomycind + Fusidicacid	Actinomycind + Fusidicacid
Amikacin + Ampicillin	Amikacin + Ampicillin
Amikacin + Cisplatin	Amikacin + Cisplatin
Amikacin + Fusidicacid	Amikacin + Fusidicacid
Amoxicillin + Fusidicacid	Amoxicillin + Fusidicacid
Ampicillin + Azithromycin	Ampicillin + Azithromycin
Ampicillin + Aztreonam	Ampicillin + Aztreonam
Ampicillin + Bacitracin	Ampicillin + Bacitracin
Ampicillin + Calcofluor	Ampicillin + Calcofluor
Ampicillin + Cccp	Ampicillin + Cccp
Ampicillin + Cisplatin	Ampicillin + Cisplatin
Ampicillin + Clarythromycin	Ampicillin + Clarythromycin
Ampicillin + EGCG	Ampicillin + EGCG
Ampicillin + Erythromycin	Ampicillin + Erythromycin
Ampicillin + Fusidicacid	Ampicillin + Fusidicacid
Ampicillin + Indolicidin	Ampicillin + Indolicidin
Ampicillin + Mecillinam	Ampicillin + Mecillinam
Ampicillin + Novobiocin	Ampicillin + Novobiocin
Ampicillin + Oxacillin	Ampicillin + Oxacillin
Ampicillin + Phleomycin	Ampicillin + Phleomycin
Ampicillin + Radicicol	Ampicillin + Radicicol
Ampicillin + Rifampicin	Ampicillin + Rifampicin
Ampicillin + Spiramycin	Ampicillin + Spiramycin
Ampicillin + Sulfamethizole	Ampicillin + Sulfamethizole
Ampicillin + Tetracycline	Ampicillin + Tetracycline
Ampicillin + Tunicamycin	Ampicillin + Tunicamycin
Ampicillin + Vancomycin	Ampicillin + Vancomycin
Ampicillin + Verapamil	Ampicillin + Verapamil
Azithromycin + Cisplatin	Azithromycin + Ceftazidime
Azithromycin + Clarythromycin	Azithromycin + Cisplatin
Azithromycin + Erythromycin	Azithromycin + Clarythromycin
Azithromycin + Fusidicacid	Azithromycin + Erythromycin
Azithromycin + Oxacillin	Azithromycin + Fusidicacid
Azithromycin + Phleomycin	Azithromycin + Oxacillin
Azithromycin + Rifampicin	Azithromycin + Phleomycin
Azithromycin + Spiramycin	Azithromycin + Rifampicin
Azithromycin + Tetracycline	Azithromycin + Spiramycin
Azithromycin + Vancomycin	Azithromycin + Tetracycline
Azithromycin + Verapamil	Azithromycin + Vancomycin
Aztreonam + Cisplatin	Azithromycin + Verapamil
Aztreonam + Fusidicacid	Aztreonam + Cisplatin
Bacitracin + Fusidicacid	Aztreonam + Fusidicacid

Bleomycin + Fusidicacid	Bacitracin + Fusidicacid
Calcofluor + Fusidicacid	Bleomycin + Fusidicacid
Carbenicillin + Fusidicacid	Calcofluor + Cisplatin
Cccp + Cisplatin	Calcofluor + Fusidicacid
Cccp + Fusidicacid	Carbenicillin + Cisplatin
Cccp + Tetracycline	Carbenicillin + Fusidicacid
Cecropinb + Fusidicacid	Cccp + Cisplatin
Cefaclor + Fusidicacid	Cccp + Fusidicacid
Cefoxitin + Fusidicacid	Cccp + Tetracycline
Cefsulodin + Fusidicacid	Cecropinb + Fusidicacid
Ceftazidime + Fusidicacid	Cefaclor + Fusidicacid
Ceftazidime + Tetracycline	Cefoxitin + Fusidicacid
Chir090 + Fusidicacid	Cefsulodin + Fusidicacid
Chloropromazine + Fusidicacid	Ceftazidime + Cisplatin
Cisplatin + Clarythromycin	Ceftazidime + Fusidicacid
Cisplatin + Fusidicacid	Ceftazidime + Tetracycline
Cisplatin + Mecillinam	Chir090 + Fusidicacid
Cisplatin + Oxacillin	Chloropromazine + Fusidicacid
Cisplatin + Phleomycin	Cisplatin + Clarythromycin
Cisplatin + Spiramycin	Cisplatin + EGCG
Cisplatin + Sulfamethizole	Cisplatin + Fusidicacid
Cisplatin + Vancomycin	Cisplatin + Indolicidin
Clarythromycin + Fusidicacid	Cisplatin + Mecillinam
Clarythromycin + Novobiocin	Cisplatin + Oxacillin
Clarythromycin + Phleomycin	Cisplatin + Phleomycin
Clarythromycin + Rifampicin	Cisplatin + Spiramycin
Clarythromycin + Spiramycin	Cisplatin + Sulfamethizole
Clarythromycin + Tetracycline	Cisplatin + Sulfamonomethoxine
Clarythromycin + Vancomycin	Cisplatin + Tetracycline
Cycloserined + Fusidicacid	Cisplatin + Vancomycin
Doxycycline + Fusidicacid	Clarythromycin + Fusidicacid
EGCG + Fusidicacid	Clarythromycin + Novobiocin
Erythromycin + Fusidicacid	Clarythromycin + Spiramycin
Fusidicacid + Glufosfomycin	Clarythromycin + Tetracycline
Fusidicacid + Hydroxyurea	Clarythromycin + Vancomycin
Fusidicacid + Indolicidin	Cycloserined + Fusidicacid
Fusidicacid + Isoniazid	Dibucaine + Fusidicacid
Fusidicacid + Mecillinam	Doxycycline + Fusidicacid
Fusidicacid + Methotrexate	EGCG + Fusidicacid
Fusidicacid + Minocycline	Erythromycin + Fusidicacid
Fusidicacid + Nigericin	Fusidicacid + Glufosfomycin
Fusidicacid + Novobiocin	Fusidicacid + Hydroxyurea
Fusidicacid + Oxacillin	Fusidicacid + Indolicidin
Fusidicacid + Phleomycin	Fusidicacid + Isoniazid
Fusidicacid + Propidiumiodide	Fusidicacid + Mecillinam
Fusidicacid + Puromycin	Fusidicacid + Methotrexate

Fusidicacid + Radicicol	Fusidicacid + Minocycline
Fusidicacid + Rifampicin	Fusidicacid + Nigericin
Fusidicacid + Spectinomycin	Fusidicacid + Novobiocin
Fusidicacid + Spiramycin	Fusidicacid + Oxacillin
Fusidicacid + Sulfamethizole	Fusidicacid + Phleomycin
Fusidicacid + Sulfamonomethoxine	Fusidicacid + Propidiumiodide
Fusidicacid + Tetracycline	Fusidicacid + Puromycin
Fusidicacid + Triclosan	Fusidicacid + Radicicol
Fusidicacid + Vancomycin	Fusidicacid + Rifampicin
Fusidicacid + Verapamil	Fusidicacid + Spectinomycin
Novobiocin + Tetracycline	Fusidicacid + Spiramycin
Oxacillin + Tetracycline	Fusidicacid + Sulfamethizole
Oxacillin + Vancomycin	Fusidicacid + Sulfamonomethoxine
Phleomycin + Tetracycline	Fusidicacid + Tetracycline
Spiramycin + Tetracycline	Fusidicacid + Triclosan
Spiramycin + Vancomycin	Fusidicacid + Vancomycin
Tetracycline + Vancomycin	Fusidicacid + Verapamil
	Oxacillin + Tetracycline
	Oxacillin + Vancomycin
	Phleomycin + Tetracycline
	Spiramycin + Tetracycline
	Tetracycline + Vancomycin
