# Supplementary material for: Chemogenomic model identifies synergistic drug combinations robust to the pathogen microenvironment
Source: PLoS Comput Biol. 2018 Dec 31;14(12):e1006677. doi: 10.1371/journal.pcbi.1006677 (PMC6329523; doi:10.1371/journal.pcbi.1006677)
Supplement: S1 Text — (PDF) [file pcbi.1006677.s020.pdf]

Contents in supplementary dataset (provided in zip file)

- MAGENTA_supplementary_dataset.xlsx –this file contains the drug interaction training and validation data
  - the sheet 'triplet predictions' contains the experimental drug interaction validation data for 56 three-way combinations
  - the sheet 'media predictions' contains the experimental drug interaction training data for MAGENTA
  - the sheet 'media predictions test' contains the experimental drug interaction validation data for 55 two-way combinations measured in glycerol media
  - the sheet 'acinetobacter' contains the experimental drug interaction validation data for 15 two-way combinations in three conditions – LB, glucose and glycerol for *A. baumannii*
- ecoli_acinetobacter_orthologs.xlsx – this file contains the list of orthologs between *E. coli* and *A. baumannii*
- MAGENTA_tutorial.m – this MATLAB file reproduces all the results in the manuscript

The raw experimental data used in our study are provided in the following files -

- Ecoli_screens.xlsx - All pairwise interactions among 8 drugs in 2 conditions, corresponding to 16 384-well microplates (Displayed in Figure 1).

- Ecoli-triplets.xlsx - All 3-way interactions among 8 drugs, corresponding to 4 microplates (Displayed in Figure 2).

- Ecoli-M9Glycerol.xlsx - All pairwise interactions among 11 drugs in glycerol media, corresponding to 10 microplates (Displayed in Figure 3).

- Abaumannii_screens.xlsx - All pairwise interactions among 6 drugs in three different media in A. baumannii, corresponding to 6 microplates (Displayed in Figure 4).
